# Artificial intelligence and social accountability in the Canadian health care landscape: A rapid literature review

**DOI:** 10.1371/journal.pdig.0000597

**Published:** 2024-09-12

**Authors:** Alex Anawati, Holly Fleming, Megan Mertz, Jillian Bertrand, Jennifer Dumond, Sophia Myles, Joseph Leblanc, Brian Ross, Daniel Lamoureux, Div Patel, Renald Carrier, Erin Cameron

**Affiliations:** 1 Dr. Gilles Arcand Centre for Health Equity, NOSM University, Thunder Bay/Sudbury, Ontario, Canada; 2 Clinical Sciences Division, NOSM University, Sudbury/Thunder Bay, Ontario, Canada; 3 Health Sciences North, Sudbury, Ontario, Canada; 4 NOSM University, UME Learner, Sudbury/Thunder Bay, Ontario, Canada; 5 Health Sciences Library, NOSM University, Sudbury/Thunder Bay, Ontario, Canada; 6 School of Sociological and Anthropological Studies, University of Ottawa, Ottawa, Ontario, Canada; 7 School of Kinesiology and Health Sciences, Laurentian University, Sudbury, Ontario, Canada; 8 Human Sciences Division, NOSM University, Sudbury/Thunder Bay, Ontario, Canada; 9 Medical Sciences Division, NOSM University, Sudbury/Thunder Bay, Ontario, Canada; National Tsing-Hua University: National Tsing Hua University, TAIWAN

## Abstract

**Background:**

Situated within a larger project entitled “Exploring the Need for a Uniquely Different Approach in Northern Ontario: A Study of Socially Accountable Artificial Intelligence,” this rapid review provides a broad look into how social accountability as an equity-oriented health policy strategy is guiding artificial intelligence (AI) across the Canadian health care landscape, particularly for marginalized regions and populations. This review synthesizes existing literature to answer the question: How is AI present and impacted by social accountability across the health care landscape in Canada?

**Methodology:**

A multidisciplinary expert panel with experience in diverse health care roles and computer sciences was assembled from multiple institutions in Northern Ontario to guide the study design and research team. A search strategy was developed that broadly reflected the concepts of social accountability, AI and health care in Canada. EMBASE and Medline databases were searched for articles, which were reviewed for inclusion by 2 independent reviewers. Search results, a description of the studies, and a thematic analysis of the included studies were reported as the primary outcome.

**Principal findings:**

The search strategy yielded 679 articles of which 36 relevant studies were included. There were no studies identified that were guided by a comprehensive, equity-oriented social accountability strategy. Three major themes emerged from the thematic analysis: (1) designing equity into AI; (2) policies and regulations for AI; and (3) the inclusion of community voices in the implementation of AI in health care. Across the 3 main themes, equity, marginalized populations, and the need for community and partner engagement were frequently referenced, which are key concepts of a social accountability strategy.

**Conclusion:**

The findings suggest that unless there is a course correction, AI in the Canadian health care landscape will worsen the digital divide and health inequity. Social accountability as an equity-oriented strategy for AI could catalyze many of the changes required to prevent a worsening of the digital divide caused by the AI revolution in health care in Canada and should raise concerns for other global contexts.

## Author summary

Social accountability has emerged over the past few decades as an equity-oriented health policy strategy in health care leading to a number of positive impacts. Given the rapid onset of an artificial intelligence (AI) revolution in health care our research team at the Dr. Gilles Arcand Centre for Health Equity wanted to broadly explore how a social accountability strategy, and linked health equity concepts, are being used to guide artificial intelligence across the Canadian health care landscape. This study summarizes the key themes of a rapid review of literature that reflect (1) the need to design equity into AI technologies; (2) the urgency for policies and regulations for AI; and (3) the inclusion of community voices in the implementation of AI in health care. The concepts of equity, marginalized populations, and engagement with community and partners were also identified across all 3 themes, which reflect key components of a social accountability strategy. An intentional, equity-oriented social accountability strategy is therefore proposed as a means to guide AI in the Canadian health care landscape, and in other global contexts, before this revolution worsens the digital divide and health inequity.

## Introduction

At a time where the digital divide has yet to be conquered, artificial intelligence (AI) has emerged as a revolutionary technology in health care [1,2]. AI is increasingly embedded in existing systems like telehealth and electronic medical records (EMRs) while also disrupting health care with precision medicine, workflow, and diagnostic skills optimization among other promising advances [3–5]. Alongside AI’s revolutionary impact in health care is the growing concern for continued compassion deficits or indifference towards people, regions, and communities that are already marginalized and affected by the digital divide and health inequities [6–9]. For example, Northern Ontario is one of many marginalized regions in Canada that experiences a continued compassion deficit driving multiple health inequities [10–15]. An indifference towards already marginalized regions in Canada, like Northern Ontario or other similar regions globally, threatens to continue historic compassion deficits that can sideline these regions from the benefits of the AI revolution in health care.

Intentionally incorporating empathy into AI technologies can counter the consequences of compassion deficits that worsen the digital divide and health inequities of marginalized people and communities [8]. The digital divide in Canada reflects structural inequities such as socioeconomic and geographic differences in access to technology between communities and populations [16]. It is most pronounced in rural, remote, and Indigenous communities where digital health literacy, access to the internet and required resources are significantly limited when compared to urban centers [17,18]. Literature across the AI and machine learning sector has only just begun to recognize geographic and population diversity, local disparities and the need to overcome the digital divide through co-creation of digital solutions with communities and key partners [19,20]. There is a critical need for a renewed approach to AI technologies in health care particularly since this technology is already being criticized for discriminating based on structural inequities such as race [21].

Emphasizing the need for AI to be accountable to society—or guided by social accountability—can be an intentional strategy that allows for compassion, empathy, and for solutions that address structural inequities supporting the digital divide [8,22]. Social accountability is a health equity strategy pioneered by the World Health Organization (WHO) [23] that has shaped health professional education and health care systems across Canada and globally with multiple positive impacts [24–33]. Social accountability strategies [23,34] are comprehensive across education, research, and service activities; reflect an obligation to address the priority health concerns within a defined region; and, are guided by the values of relevance, quality, cost-effectiveness, and most importantly equity. It is achieved through partnerships centering on authentic, empowered community engagement—particularly with those who are marginalized, underserved, and who experience inequity [34–36].

There is an opportunity to intentionally prioritize compassion, empathy, and the accountability of AI systems for people and communities who are negatively impacted by the digital divide and other structural forms of health inequity [37]. The objective of this study is to understand how AI is present and impacted by social accountability across the health care landscape in Canada. To realize this objective, a rapid review of literature was completed.

## Results

### Search results

**Fig 1:**
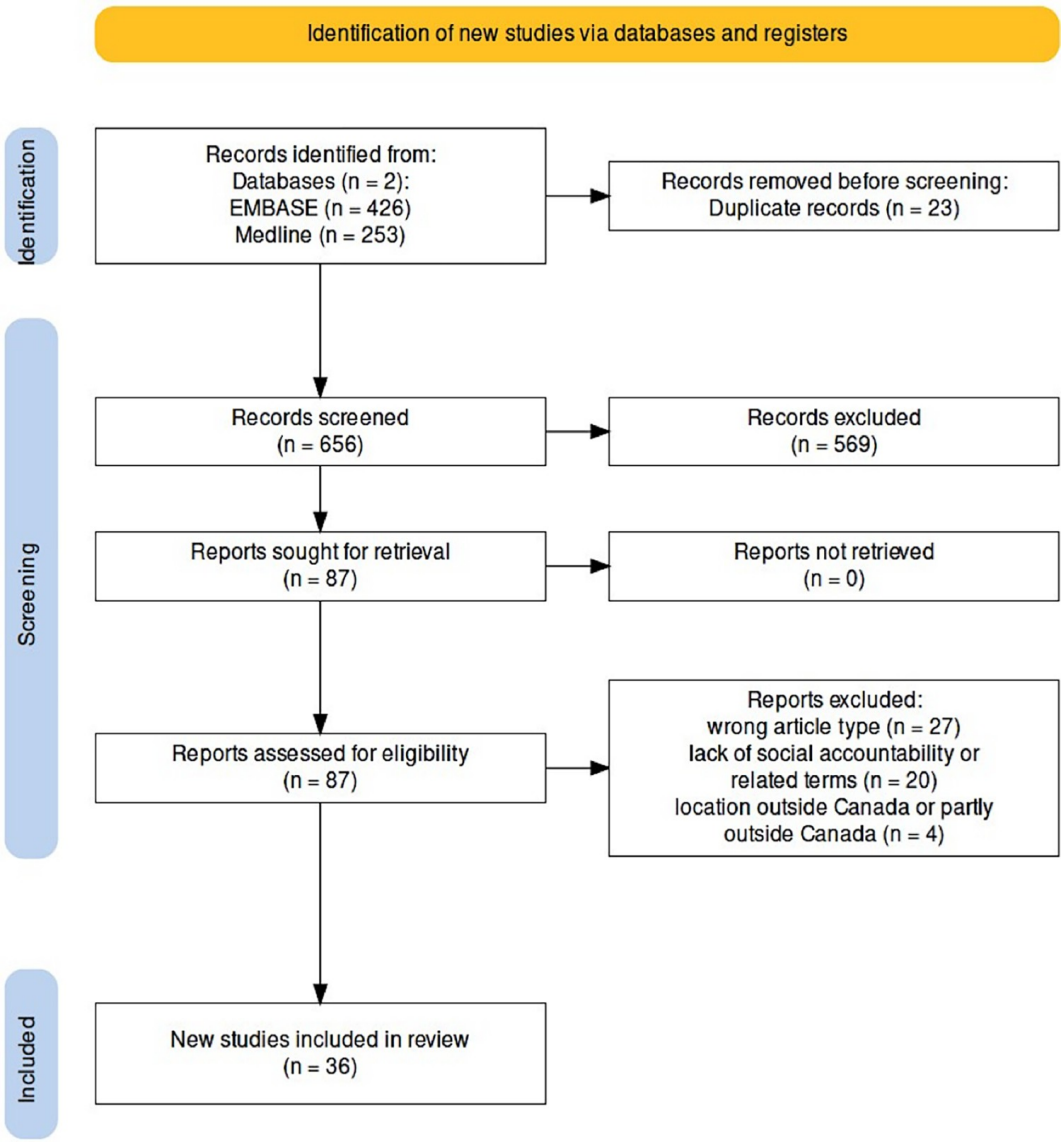
PRISMA-like flow chart.

Summary of the search findings and article selection is outlined in **Fig 1**. A total of 679 (Ovid EMBASE, *n* = 426; Ovid MEDLINE, *n* = 253) articles were identified for screening; 36 met the inclusion criteria following the removal of duplicates, title, abstract and citation screening, and full-text review.

### Study characteristics

Summary of key descriptions of the included studies are listed in **Table 1**. Comprehensive study characteristics are listed in **S1 File**. Excluding telemedicine, e-health, and EMR focused studies, very few studies (*n* = 2) specifically addressed novel AI technologies in health care such as machine learning. The majority of the studies were in Ontario (*n* = 18), focused on health care service delivery (*n* = 33) and addressed the micro (health care provider-patient) (*n* = 11) and meso (local health care and community environment) (*n* = 19) levels. A minority of studies focused exclusively on rural and remote areas (*n* = 4), Indigenous communities (*n* = 2), few addressed AI in health research (*n* = 3), none were identified in health professional education and few focused on the macro (political and policy) (*n* = 6) level.

**Table 1:** Summary description of included studies.

Study characteristic	Number of studies
*Methodology*	
Quantitative	8
Qualitative	20
Mixed methods	8
*Location*	
Canada (national focus) Multi-Province	8 2^!^
Ontario (total) Ontario (entire province) Southern Ontario (total) Toronto Rural Southern Windsor-Essex region Eastern Ontario (total) Eastern Ontario (entire region) Kingston Champlain region Northern Ontario (total)	*18* 6 5 3 1 1 6 2 1 3 1
British Columbia	4
Alberta	1
Quebec	1
Nunavut	1
Manitoba	1
*Study focus* ^*^	
Rural/remote communities	4
Indigenous communities	2
*References impact on* ^*#*^	
Rural/remote communities^*^	19
Indigenous communities^*^	10
*Health care domain*	
Health care service delivery	33
Health research	3
Health professional education	0
*System level*	
Micro (patient–health worker interactions)	11
Meso (local health care and community environment)	19
Macro (political and policy contexts)	6

^!^Includes 1 study in Ontario and Quebec and 1 study in Ontario, Alberta, BC and Quebec.

*Represents studies with an intentional and clear focus on rural/remote or Indigenous communities.

^#^Represents studies that did not intentionally focus on rural/remote or Indigenous communities, but briefly reference possible impacts.

### Thematic analysis

Three major themes linked to how AI is present and impacted by social accountability emerged from the included studies—(1) designing equity into AI technologies; (2) policies and regulations for AI; and (3) including community voices during the implementation. Additionally, equity, marginalized populations, and community and partner engagement were consistently identified as key concepts. The thematic analysis summary can be found in **S2 File**.

#### Designing equity into AI technologies

The first theme emphasized the need to design equity into AI technologies for health care [38–40] to address a digital divide that is driven by structural inequities [38,39,41,42]. Studies identified several considerations for designing equity into AI technologies including perceived benefits, anticipating intervention-generated inequity, focusing on digital literacy, addressing structural inequities, addressing access barriers for marginalized populations, and the critical need to engage with communities and other partners.

Designing equity into AI technologies has benefits. eConsult services and access to telemedicine [38,40,43–53], for example, can reduce travel time and costs and improve access to specialist care for people from rural communities, with lower socioeconomic backgrounds, transgender and nonbinary patients, and residents of long-term care facilities [40,46,53–56].

There is a risk of intervention-generated inequality unintentionally designed into AI technologies [57]. Virtual care, for example, tends to benefit those with the fewest barriers in accessing the technology; while it risks perpetuating inequity and the digital divide for marginalized groups who face unrecognized access barriers [38,41,43,54]. One study by Moroz and colleagues proposed that the design of AI technologies such as virtual care should be strengthened through redesign in this context [54,58].

Several studies described the need for an intensified focus on digital literacy in designing AI [38–40,43,54,57,59,60]. The importance of digital literacy was described in relation to virtual care and assumptions of acceptance by all patients for AI supported technology such as eConsults [38,40]. One study noted that even technologically competent patients experienced difficulty utilizing telehealth, which impacted patient care by reducing the total time spent in appointments [38]. Rush and colleagues describe that addressing equity for rural communities through digital literacy “…will need to involve telemedicine literacy training and support.” [60]. Poor digital literacy was also linked to equity in studies that described the negative impacts for populations with lower socioeconomic backgrounds, older patients, patients with disabilities, and refugee and immigrant populations [38,39,42,59].

Multiple studies identified structural inequities that need to be addressed in AI design [41,42,61]. Several studies noted that the basic prerequisite of a computer and internet connectivity was frequently overlooked [38,39,57]. Referencing the digital divide, Gidhei and colleagues [42] describe “the inequitable access to internet and technology, socioeconomic barriers, language barriers, low literacy levels, and limited access to virtually delivered interventions and services” limit access to AI technologies [42]. While another study noted “financial costs, but also cultural and structural obstacles: low income; unemployment; racial discrimination; literacy; housing; social exclusion; stigma; perceptions of health, mental health, and services; and linguistic barriers…” [59] as structural inequities refugees face accessing virtual mental health services.

Several studies further noted that people who are marginalized, with lower incomes, those experiencing homelessness or refugees, should be substantially engaged in AI designs [38,39,57]. When marginalized communities are considered in the design process, the benefits of AI technologies have the greatest impact [38,39,43,54,57]. Lam and colleagues [61] ironically report that patients with lower socioeconomic status, who tend to experience greater comorbidities, would benefit substantially from the convenience of follow-up via telehealth, which can be inaccessible to them due to the strained financial resources required to access the internet, personal communication devices, or reliable mobile phone data plans. It would appear to be that those who can benefit the most from AI technologies are people and communities experiencing some form of marginalization and should be prioritized for engagement [38–42,52,57,58,61]. Community and partner engagement in the design process would ensure local values and core needs are recognized and respected [38,52,57].

#### Policies and regulations for AI in health care

The second theme emerged from 12 studies that underscored the need for policies and regulations that can govern the use and influence of AI in health care [38,46,52,58,59,62–67]. These studies highlighted key concepts for AI policy and regulation development including equity as a central policy value and data management approaches that define the use, collection, storage, protection, access, and data sovereignty.

The importance of equity was highlighted as a central value to the policies and regulations for the use of AI in health care. For example, Shaid and colleagues suggested that to maximize equity in AI policies it will be necessary to consider the individual, technological, health system, and social determinants of health levels [38]. Additionally, Ghidei and colleagues described a methodology to achieve equity in AI policies as requiring an anti-colonial and antiracist perspective along with the inclusion of diverse perspectives from Indigenous and other racialized populations [42]. Ghidei and colleagues further discussed the importance of accounting for systemic and institutional-level barriers experienced by marginalized communities in AI policies [42]. At a systems and social determinants of health level, several studies noted that both federal and provincial governments need to consider policy choices that allocate resources, funding, and subsidies that can narrow the digital divide [38,39,42,43,54,57,59,60]. Different levels of government can collaborate to improve equitable access to AI technologies in health care, for example, by allocating funds to enhance broadband high-speed internet access in rural communities and establishing programs that can enhance digital literacy or subsidize cellular phone service [38,42,60].

AI technologies use and generate large amounts of data highlighting the critical need for data management policies and regulations that address the use, collection, storage, protection, access, and sovereignty of data particularly for diverse and vulnerable populations. Four studies discussed accessing data obtained through EMR to analyze patient demographics [52,64–66]. These studies analyzed data for migrant workers, racialized individuals, and other marginalized groups resulting in recommendations aimed at improving care noting that the use of EMR data yielded high-quality information [52,64–66]. As another example, Kundu and colleagues [67] employed machine learning and random forest modeling to predict the risk of past-year suicidal thoughts among sexual and gender minority young adults with findings that could influence targeted, mental health support and suicide prevention for the LGBTQI2S+ population [67].

Despite these promising advantages of allowing access to EMR data for AI technologies, patient concerns with privacy, data protection, and subsequent harm when accessing AI technologies such as virtual care were identified [38,44,45,52,53,59,61,63]. Studies underscored the importance that AI data management and protection policies must account for factors such as privacy, licensing, and regulatory variations across different jurisdictions [38,44,52,58,63]. Paprica and colleagues identified 12 criteria for the timely, safe, and equitable handling of public health data that organizations could follow [63]. Data sovereignty was also highlighted by Wilkinson and colleagues [52] in reference to Indigenous communities who often have notable gaps in the availability of health data. Indigenous data sovereignty recognizes that data has been stolen through the processes of residential schools, the Indian Act and similar anti-Indigenous initiatives and recognizes Indigenous people’s right to govern how their data is used [42,52,68]. Wilkinson and colleagues [52] raised several recommendations for Indigenous data sovereignty:

Clarity regarding rights of ownership, control over, conditions of access, and possession of health information were important achievements in this EMR implementation. It is therefore a recommendation that privacy and confidentiality matters be introduced early in the planning processes for HIS [hospital information systems] in First Nations communities and adequate resources and discussion time should be dedicated for these discussions.

#### Inclusion of community voices during the implementation of AI in health care

The third theme reflects the inclusion of community voices during the implementation of AI technologies in health care. Inclusive AI implementation refers to the empowerment of diverse people and marginalized communities in the implementation of AI to provide critical insights that will maximize their benefit from AI technologies rather than perpetuate harm and inequity. Overall, 13 studies [38,40,42,44–47,52,53,58,59,67,69] discussed barriers, facilitators, and considerations for diverse, marginalized populations for the implementation of AI technologies [38,42,43,59,60].

There were multiple barriers identified for the implementation of AI technologies. Five studies discussed the need to address systemic and institutional barriers [38,42,43,59,60]. Apprehension and reluctance by patients and providers and privacy concerns contribute to challenging transitions to AI technologies such as telehealth and virtual care [53,60]. Similarly, forced, unsupported, rapid implementation, as was the case during the COVID-19 pandemic, was described as another challenge transitioning to these technologies [17,46,70,71]. Language and culture were reported as a barrier to a patient’s ability to fully express their concerns [38,59,64,65,69]. Culturally unsafe spaces for accessing AI technologies such as virtual care were also seen as a barrier [42,52,59]. Additionally, implementing AI technologies tends to burden communities with adapting to changes in the delivery of health care services, with one study specifically reporting a lack of communication with the community as a barrier [38,59,67].

There were also a number of notable facilitators for the implementation of AI-influenced technologies. One study described using the “Best Practices for EHR Implementation Framework” for the successful implementation of EMR [52]. More favorable treatment outcomes were noted with patient and provider satisfaction alignment with the technology being used [44,49,53]. Culturally safe, trauma-informed methods of implementation were facilitators [38,42,59,69]. Anticipating considerations about patient privacy, reimbursement models, new workflows, IT support, and staff resourcing also facilitate implementation [38,46,52]. Although described as a barrier as well, the need for rapid implementation during the COVID-19 pandemic was a facilitator for technologies such as virtual care [49,50,52–55,61].

A thorough understanding of the problem that an AI technology addresses from multiple, diverse perspectives was also identified as an implementation facilitator. Liddy and Keely highlight this stating:

Health technology solutions are too often implemented without a true understanding of the system-level problem they seek to address, resulting in excessive costs, poor adoption, ineffectiveness, and ultimately failure. Before implementing or adopting health care innovations, partners should complete a thorough assessment to ensure effectiveness and value [40].

Other studies further identified key partners for active engagement in implementation [59]. “Implementation of [virtual care] needs to be conceptualized from the perspectives of all relevant partners, including clients, therapists, the operational frameworks of organizations, the larger health systems, funders, and policy makers.” [59]. One study investigating EMR implementation at a First Nations health center concluded that “provincial and federal government commitment and collaboration with key partners, including a local physician champion, were critically important …” [52]. Several studies discussed the need to involve community members as partners during implementation to ensure appropriate care for diverse and marginalized communities [38,42,58,59,69].

Several marginalized populations were identified in a number of studies as being negatively impacted through the implementation of AI technologies. Ineffective communication about changes to access led some marginalized populations to believe services were stopped altogether [38]. Linguistic and cultural minority populations experience challenges [38,59,64,65,69]. Indigenous and immigrant populations were apprehensive to share experiences in culturally unsafe virtual spaces [42,52,59]. Survivors of intimate partner violence can experience re-traumatization when accessing AI technologies in the same, unsafe space where their trauma occurred [42]. The rapid adoption of virtual care during the COVID-19 pandemic had negative impacts on diverse, marginalized populations and did not allow for the time nor resources to maximize equity [38,39,41,42,54,57,59–62]. Indigenous and rural residents were referenced as marginalized populations in several studies [17,18,38,44,52,55,57,61,71–80]. First Nations participants in one study “did not feel that virtual care was implemented in a way that resonated with their cultural beliefs about health, wellness, and healing.” [38].

## Discussion

### Main outcomes

There were 3 notable findings from this rapid review of literature that help understand how AI is present and impacted by social accountability across the health care landscape in Canada. The findings include (1) the need to design greater equity into AI; (2) the urgent need for policies and regulations for AI; and (3) including community voices during the implementation. Across these 3 main findings equity, marginalized populations and the need for community and partner engagement were frequently referenced concepts.

The descriptive characteristics of the reviewed studies also illustrate a number of notable findings. The impact of social accountability and linked health equity concepts on AI in health care remains relatively underdeveloped in Canada. No studies explicitly referenced using a comprehensive, equity-oriented social accountability strategy to guide AI for health care. However, multiple studies referenced core components and values central to social accountability strategies such as equity, the need to focus on marginalized populations and engagement with community and partners in the co-creation of AI technologies. Multiple studies mention rural and remote communities (*n* = 19) as well as Indigenous communities (*n* = 10) to varying degrees. Despite the awareness, only a small fraction of studies were intentionally focused on rural and remote (*n* = 4) and Indigenous communities (*n* = 2), which are considered marginalized regions and populations in Canada. Although not the only focus of this rapid review, it is also telling that there was only one study that included Northern Ontario. Overall, these findings suggest that there is a need for an intentional, equity-oriented social accountability strategy to redirect AI in health care towards bridging what appears to be a worsening of the digital divide and compassion deficits towards marginalized regions and populations who experience health inequity.

### Limitations

This study was limited by a rapid review methodology, which was chosen to concurrently inform the AI-North community of practice. Gray literature was not included, which could yield additional findings. Translating search concepts into search terms was also challenging. For example, the expert panel advised the use of a broad definition for AI that included readily identifiable AI technologies such as machine learning, but also telehealth, virtual care, and EMR which can have varying degrees of AI programming [3,5]. Lastly, articles were selected from the Canadian context, which may not translate well to other global contexts dependent on local geography, health care system administration, social policies, and population demographics.

### Strengths

The study’s design included a diverse, expert, multidisciplinary advisory panel grounded in Northern and rural Canadian contexts. The methods were designed for rigor and reproducibility. The search strategy, critical search concepts, and terms were kept broad to capture the breadth and depth of social accountability and AI in health care. The thematic analysis facilitated the practical mobilization of knowledge for the AI-North community of practice.

### Comparison with other studies

There are no studies that apply the search concepts of social accountability and AI in the context of the Canadian health care landscape. There does exist literature that also recognizes a worsening digital divide, geographic diversities, local disparities, and the need to co-create digital solutions with communities and key partners [19,20].

### Future research

Beyond the Canadian contexts, the results of this study should signal the urgent need to critically appraise the trajectory of the AI revolution in health care in other global contexts. Future studies can build on these findings and explore the impact of AI across other marginalized populations and communities; the use of social accountability as an equity-oriented health policy strategy for AI in health care; and, alternative equity frameworks for AI.

### Conclusions

Accounting for the limitations and strengths, the findings suggest that unless there is a course correction, AI in the Canadian health care landscape is poised to worsen the digital divide and health inequity for regions like Northern Ontario. This should also raise concern for the trajectory of AI in health care in other global contexts. AI developers must consider the importance of designing greater equity into AI, the urgent need for policies and regulations, and including community voices during the implementation of AI technologies. This reflects the importance of centering on equity, prioritizing marginalized populations and engaging with communities and partners in their co-design. Based on these findings, social accountability as an equity-oriented strategy for AI could provide the catalyst to overcome a worsening of the digital divide brought on by the AI revolution in health care.

## Materials and methods

### Study design

This study is motivated by the health inequities [12–15] experienced in one of Canada’s marginalized regions—Northern Ontario, which like other regions in Canada and globally, requires a health equity strategy [10] that also extends to AI in health care. This rapid review of literature, as part of a broader study titled “Exploring the Need for a Uniquely Different Approach in Northern Ontario: A Study of Socially Accountable Artificial Intelligence,” seeks to provide an overview of how the emergence of social accountability as an equity-oriented health policy strategy is present and impacts AI across the Canadian health care landscape, particularly for marginalized regions and populations. The Canadian health care system aims to provide publicly funded “universally accessible” health care, but struggles with these objectives [81].

A rapid review methodology was chosen for the rapid translation of knowledge [71,74,75,82] to inform the AI-North community of practice. The rapid review protocol was developed a priori, registered with Open Science Framework (https://doi.org/10.17605/OSF.IO/URE7V), completed between February and October 2023 and was guided by an expert advisory panel.

### Expert advisory panel

A 12 person expert advisory panel consisting of educators, physicians, learners, and researchers oversaw the study design; directed the activities of the research team; developed the research protocol; defined key concepts and terms for the search strategy; reviewed the inclusion/exclusion criteria for study selection; and reviewed data extraction, synthesis, and results [76]. The advisory panel included members from the Dr. Gilles Arcand Centre for Health Equity at NOSM University, Lakehead University and the SAFE for Health Institutions Project in Northern Ontario, Canada with expertise in social accountability, health equity and AI in health professional education, health care service delivery, health system administration, health research, and computer sciences disciplines.

### Research team

The research team consisted of the study’s leads (AA and EC), 3 research assistants (HF, MM, and JB), and a health sciences librarian (JD). AA is an emerging, physician clinical researcher and health care leader, while EC is a mid-career health professional education researcher; both bring expertise on the application of social accountability in health care.

### Search strategy

EMBASE (Ovid) and MEDLINE (Ovid) databases were searched for peer reviewed, primary literature published between 2018 and 2023 to capture the recent, rapid evolution of AI in health care. The search strategy was focused on: (1) social accountability and linked health equity concepts; (2) AI technologies; (3) Canada; and (4) health care. The expert advisory panel directed the research team to prioritize broad, inclusive search terms in 3 key areas of the search strategy. The first was to include health equity terminology that can be linked to the breadth, depth, and expectations of a social accountability strategy in health care (i.e., antiracism, community engagement). The second was to consider all forms of AI embedded into existing technologies, such as virtual care, EMR and telemedicine, as well as, new AI systems and technologies in novel fields such as machine learning and robotics among others [83]. The third was to consider the health care landscape in Canada, beyond Northern Ontario, to include health professional education, health care service delivery, and health research. The search strategy was limited to Canadian studies to maximize the external validity of the results when considering the impact of differing geographies, health care systems, social policies, and population demographics in other global contexts. The search strategy was finalized with a health sciences librarian and executed in September 2023 (**S3 File**).

### Article screening

Reviewers were tasked with identifying articles that addressed the intersection of social accountability and AI in the context of Canada’s health care landscape. Using Covidence, 2 reviewers (MM and JB) independently screened for inclusion (**Table 2**) article titles, abstracts and citations, and retrieved full-texts for review resolving conflicts first by consensus and then with a third reviewer (HF). Full-text articles were assessed for quality using agreed upon criteria related to (1) relevancy; (2) reliability; (3) validity; and (4) applicability (**S4 File**).

**Table 2:** Inclusion and exclusion criteria for article selection.

Inclusion criteria	Exclusion criteria
Context ◾Health professional education ◾Health care service delivery ◾Health research	
Concept ◾AI technologies ◾*AND* ◾Social accountability or related health equity concepts	**Concept** ◾Does not explicitly reference AI technologies
Population/location ◾Canada	**Population/location** ◾Outside of Canada
Article types ◾Any language translated to English ◾Fully accessible online ◾Published 2018–2023 ◾Passes quality appraisal	**Article types** ◾Commentaries/editorials ◾Conference abstract ◾Letters to the editor ◾Non-article sources: books, book chapters ◾Duplicate articles

### Data extraction and synthesis

#### Study characteristics

Authors, publication year, title, journal, study location, study type (qualitative, quantitative, mixed methods), design/methodology, population, objective, AI technology, and conclusions were extracted from the included studies. Articles were further categorized based on the domains of health professional education, health care service delivery, health research and whether they were focused on the micro (health care provider-patient), meso (local health care and community environment), or macro (political and policy contexts) levels.

#### Thematic analysis

Two reviewers (HF and MM) conducted an inductive thematic analysis, similar to the process suggested by Wilson and colleagues [76] to identify themes from the included articles. Themes were cross referenced with the domains of health professional education, health care service delivery, health research as well as micro, meso, macro levels to identify any additional themes from the data.

#### Outcomes

The 3 primary outcomes reported include (1) the search results; (2) a description of the included studies; and (3) a narrative of the thematic analysis describing how AI is present and impacted by social accountability across the Canadian health care landscape.

## Supporting information

S1 File Comprehensive study characteristics of included studies. (PDF)

S2 File Thematic analysis. (XLSX)

S3 File Search strategy. (PDF)

S4 File Quality appraisal. (PDF)

## References

[1] Briganti G, Le Moine O. Artificial Intelligence in Medicine: Today and Tomorrow. Front Med (Lausanne) [Internet]. 2020;7. Available from: https://www.frontiersin.org/articles/10.3389/fmed.2020.00027. doi: 10.3389/fmed.2020.00027 32118012 PMC7012990

[2] Kassam A, Kassam N. Artificial intelligence in healthcare: A Canadian context. Healthc Manage Forum [Internet]. 2020;33(1):5–9. doi: 10.1177/0840470419874356 31505963

[3] Hamet P, Tremblay J. Artificial intelligence in medicine. Metabolism [Internet]. 2017;69:S36–S40. Available from: https://www.sciencedirect.com/science/article/pii/S002604951730015X. doi: 10.1016/j.metabol.2017.01.011 28126242

[4] Amisha, Malik P, Pathania M, Rathaur VK. Overview of artificial intelligence in medicine. J Family Med Prim Care [Internet]. 2019;8(7):2328–31. Available from: https://www.ncbi.nlm.nih.gov/pmc/articles/PMC6691444/. doi: 10.4103/jfmpc.jfmpc_440_19 31463251 PMC6691444

[5] Mintz Y, Brodie R. Introduction to artificial intelligence in medicine. Minim Invasive Ther Allied Technol [Internet]. 2019;28(2):73–81. doi: 10.1080/13645706.2019.1575882 30810430

[6] Keskinbora KH. Medical ethics considerations on artificial intelligence. J Clin Neurosci [Internet]. 2019;64:277–282. Available from: https://www.sciencedirect.com/science/article/pii/S0967586819300256. doi: 10.1016/j.jocn.2019.03.001 30878282

[7] Privacy Murdoch B. and artificial intelligence: challenges for protecting health information in a new era. BMC Med Ethics [Internet]. 2021;22(1):122. doi: 10.1186/s12910-021-00687-3 34525993 PMC8442400

[8] Srinivasan R, San Miguel González B. The role of empathy for artificial intelligence accountability. J Responsible Technol [Internet]. 2022;9:100021. Available from: https://www.sciencedirect.com/science/article/pii/S2666659621000147.

[9] Choudhury A, Asan O. Impact of accountability, training, and human factors on the use of artificial intelligence in healthcare: Exploring the perceptions of healthcare practitioners in the US. Hum Factors Healthcare [Internet]. 2022;2:100021. Available from: https://www.sciencedirect.com/science/article/pii/S2772501422000185.

[10] Health Quality Ontario. Northern Ontario Health Equity Strategy [Internet]. 2017 [cited 2021 Nov 1]. Available from: https://www.hqontario.ca/What-is-Health-Quality/Health-Equity-and-Quality/Our-Work/Northern-Ontario-Health-Equity-Strategy.

[11] Office of the Auditor General of Ontario. Value-for-Money Audit: Hospitals in Northern Ontario Delivery of Timely and Patient-Centred Care. Ontario Ministry of Health, Ontario Health [Internet]. 2023 Dec [cited 2024 Jul 2]. Available from: https://www.auditor.on.ca/en/content/annualreports/arreports/en23/AR_hospitalsnorth_en23.pdf.

[12] Newbery S. Physician Workforce Strategy [Internet]. 2023 Jun [cited 2024 Jul 2]. Available from: https://www.nosm.ca/our-community/nosm-physician-workforce-strategy/.

[13] Wood B. Considering equity in public health and health systems: An example from northwestern Ontario. [Internet]. National Collaborating Centre for Determinants of Health (NCCDH). 2018. Available from: https://nccdh.ca/blog/entry/considering-equity-in-public-health-and-health-systems-an-example-from-nort

[14] King M, Malaviarachchi D, Palangio A, Lefebvre M. SDHU Population Health Profile Summary Report [Internet]. Sudbury & District Health Unit Revised. 2017. Available from: www.sdhu.com.

[15] Gomes T, Murray R, Kolla G, Leece P, Bansal S, Besharah J, et al. Changing Circumstances Surrounding Opioid-Related Deaths in Ontario [Internet]. Toronto; 2021 [cited 2024 Jul 2]. Available from: https://www.publichealthontario.ca/-/media/Documents/C/2021/changing-circumstances-surrounding-opioid-related-deaths.pdf?rev=3207bc61021c4b5d8cc48fe55ebb7e23&sc_lang=en

[16] Van Dijk JAGM. The Digital Divide. 2019.

[17] Intahchomphoo C. Indigenous Peoples, Social Media, and the Digital Divide: A Systematic Literature Review. Am Indian Cult Res J. 2018 Oct 1;42(4):85–111.

[18] Koch K. The Territorial and Socio-Economic Characteristics of the Digital Divide in Canada. Can J Reg Sci. 2022 Sep 13;45(2):89–98.

[19] Berdahl CT, Baker L, Mann S, Osoba O, Girosi F. Strategies to Improve the Impact of Artificial Intelligence on Health Equity: Scoping Review. JMIR AI. 2023 Feb 7;2:e42936. doi: 10.2196/42936 38875587 PMC11041459

[20] Hendricks-Sturrup R, Simmons M, Anders S, Aneni K, Wright Clayton E, Coco J, et al. Developing Ethics and Equity Principles, Terms, and Engagement Tools to Advance Health Equity and Researcher Diversity in AI and Machine Learning: Modified Delphi Approach. JMIR AI. 2023 Dec 6;2:e52888. doi: 10.2196/52888 38875540 PMC11041493

[21] Zuiderveen Borgesius F. Discrimination, artificial intelligence, and algorithmic decision-making. 2018. Available from: https://dare.uva.nl/search?identifier=7bdabff5-c1d9-484f-81f2-e469e03e2360.

[22] Kieslich K, Keller B, Starke C. Artificial intelligence ethics by design. Evaluating public perception on the importance of ethical design principles of artificial intelligence. Big Data Soc [Internet]. 2022;9(1):20539517221092956. doi: 10.1177/20539517221092956

[23] Boelen C, Heck J. Defining and measuring the social accountability of medical schools [Internet]. Geneva; 1995 [cited 2022 Jul 30]. Available from: https://apps.who.int/iris/handle/10665/59441.

[24] Barber C, van der Vleuten C, Leppink J, Chahine S. Social Accountability Frameworks and Their Implications for Medical Education and Program Evaluation: A Narrative Review. Acad Med [Internet]. 2020;95(12):1945–1954. doi: 10.1097/ACM.0000000000003731 32910000

[25] Rezaeian M, Pocock L. Social accountability -a challenge for global medical schools. Middle East J Family. 2011 Jan 1;9:15–19.

[26] Strasser R, Hogenbirk JC, Minore B, Marsh DC, Berry S, McCready WG, et al. Transforming health professional education through social accountability: Canada’s Northern Ontario School of Medicine. Med Teach. 2013 Jun;35(6):490–6. doi: 10.3109/0142159X.2013.774334 23496120

[27] Hogenbirk J, Timony P, French M, Strasser R, Pong R, Cervin C, et al. Milestones on the social accountability journey family medicine practice location of Northern Ontario School of Medicine graduates. Can Fam Physician. 2016 Mar;62. PMC4984600 27427565

[28] Hogenbirk JC, Robinson DR, Hill ME, Pong RW, Minore B, Adams K, et al. The economic contribution of the Northern Ontario School of Medicine to communities participating in distributed medical education. Can J Rural Med. 2015;20(1):25–32. 25611911

[29] Hogenbirk JC, Robinson DR, Strasser RP. Distributed education enables distributed economic impact: the economic contribution of the Northern Ontario School of Medicine to communities in Canada. Health Econ Rev. 2021 Dec 1;11(1). doi: 10.1186/s13561-021-00317-z 34109460 PMC8191106

[30] Woolley T, Middleton L, Kavishe B, Erio T. A social return on investment analysis of the Touch Foundation’s treat & train external clinical rotations program for medical and nursing students in the Lake Zone of Tanzania [Internet]. New York; 2019 [cited 2022 Jul 12]. Available from: https://touchfoundation.org/wp-content/uploads/2019/04/2019.04.04-TT-Evaluation_long.pdf.

[31] Woolley T, Larkins S, Sen Gupta T. Career choices of the first seven cohorts of JCU MBBS graduates: Producing generalists for regional, rural and remote northern Australia. Rural Remote Health. 2019 Apr 1;19(2). doi: 10.22605/RRH4438 30943751

[32] Woolley T, Cristobal F, Siega-Sur JJ, Ross S, Neusy AJ, Halili SD, et al. Positive implications from socially accountable, community-engaged medical education across two Philippines regions. Rural Remote Health. 2018 Feb 1;18(1). doi: 10.22605/RRH4264 29453906

[33] Woolley T, Halili SD, Siega-Sur JL, Cristobal FL, Reeve C, Ross SJ, et al. Socially accountable medical education strengthens community health services. Med Educ. 2018 Apr 1;52(4):391–403. doi: 10.1111/medu.13489 29266421

[34] Anawati A, Cameron E, Harvey J. Exploring the development of a framework of social accountability standards for healthcare service delivery: a qualitative multipart, multimethods process. BMJ Open. 2023 Sep 14;13(9):e073064. doi: 10.1136/bmjopen-2023-073064 37709334 PMC10503373

[35] Boelen C. Challenges and opportunities for partnership in health development a working paper [Internet]. Geneva; 2000 [cited 2022 Jul 30]. Available from: https://apps.who.int/iris/handle/10665/66566?search-result=true&query=Challenges+and+Opportunities+for+Partnership+in+Health+Development%3A+A+Working+Paper&scope=&rpp=10&sort_by=score&order=desc.

[36] Markham R, Hunt M, Woollard R, Oelke N, Snadden D, Strasser R, et al. Addressing rural and Indigenous health inequities in Canada through socially accountable health partnerships. BMJ Open [Internet]. 2021 Nov 22;11(11):e048053. Available from: https://bmjopen.bmj.com/lookup/doi/10.1136/bmjopen-2020-048053. 34810181 10.1136/bmjopen-2020-048053 PMC8609942

[37] Ahmmed T, Alidadi A, Zhang Z, Chaudhry AU, Yanikomeroglu H. The Digital Divide in Canada and the Role of LEO Satellites in Bridging the Gap. 2022. Available from: http://arxiv.org/abs/2203.08933.

[38] Shahid S, Hogeveen S, Sky P, Chandra S, Budhwani S, de Silva R, et al. Health equity related challenges and experiences during the rapid implementation of virtual care during COVID-19: a multiple case study. Int J Equity Health [Internet]. 2023;22(1):44. doi: 10.1186/s12939-023-01849-y 36906566 PMC10007658

[39] Bhatti S, Dahrouge S, Muldoon L, Rayner J. Using the quadruple aim to understand the impact of virtual delivery of care within Ontario community health centres: a qualitative study. BJGP Open. 2022 Dec;6(4):BJGPO.2022.0031. doi: 10.3399/BJGPO.2022.0031 36109022 PMC9904779

[40] Liddy C, Keely E. Using the Quadruple Aim Framework to Measure Impact of Heath Technology Implementation: A Case Study of eConsult. J Am Board Fam Med. 2018 May 9;31(3):445–55. doi: 10.3122/jabfm.2018.03.170397 29743227

[41] Latulippe K, Hamel C, Giroux D. Co-Design to Support the Development of Inclusive eHealth Tools for Caregivers of Functionally Dependent Older Persons: Social Justice Design. J Med Internet Res. 2020 Nov 9;22(11):e18399. doi: 10.2196/18399 33164905 PMC7683256

[42] Ghidei W, Montesanti S, Wells L, Silverstone PH. Perspectives on delivering safe and equitable trauma-focused intimate partner violence interventions via virtual means: A qualitative study during COVID-19 pandemic. BMC Public Health [Internet]. 2022;22(1):1852. doi: 10.1186/s12889-022-14224-3 36195844 PMC9530429

[43] Cao J, Felfeli T, Merritt R, Brent MH. Sociodemographics Associated With Risk of Diabetic Retinopathy Detected by Tele-Ophthalmology: 5-Year Results of the Toronto Tele-Retinal Screening Program. Can J Diabetes. 2022 Feb;46(1):26–31. doi: 10.1016/j.jcjd.2021.05.001 34144907

[44] Dean P O’Donnell M, Zhou L, Skarsgard ED. Improving value and access to specialty medical care for families: a pediatric surgery telehealth program. Can J Surg. 2019 Dec;62(6):436–41. 31782575 10.1503/cjs.005918 PMC6877391

[45] Marshall T, Viste D, Jones S, Kim J, Lee A, Jafri F, et al. Beliefs, attitudes and experiences of virtual overdose monitoring services from the perspectives of people who use substances in Canada: a qualitative study. Harm Reduct J [Internet]. 2023;20(1):80. doi: 10.1186/s12954-023-00807-9 37355610 PMC10290798

[46] Helmer-Smith M, Fung C, Afkham A, Crowe L, Gazarin M, Keely E, et al. The Feasibility of Using Electronic Consultation in Long-Term Care Homes. J Am Med Dir Assoc [Internet]. 2020;21(8):1166–1170.e2. Available from: https://www.sciencedirect.com/science/article/pii/S1525861020302486. doi: 10.1016/j.jamda.2020.03.003 32360222

[47] Carroll JC, Liddy C, Afkham A, Keely E, Goh ES, Graham GE, et al. Use of eConsult to enhance genetics service delivery in primary care: A multimethod study. Genet Med. 2022 Oct;24(10):2034–41. doi: 10.1016/j.gim.2022.07.003 35947109

[48] Collins-Fairclough A, Barn P, Hirsch-Allen AJ, Rideout K, Shellington EM, Lo W, et al. Disparities in self-reported healthcare access for airways disease in British Columbia, Canada, during the COVID-19 pandemic. Insights from a survey co-developed with people living with asthma and chronic obstructive pulmonary disease. Chron Respir Dis [Internet]. 2023;20:14799731231172518. doi: 10.1177/14799731231172518 37171831 PMC10184213

[49] Walia S, Wolfe D, Keast D, Ho C, Ethans K, Worley S, et al. Facilitators and Barriers for Implementing an Internet Clinic for the Treatment of Pressure Injuries. Telemed e-Health [Internet]. 2019;25(12):1237–1243. Available from: https://www.liebertpub.com/doi/abs/10.1089/tmj.2018.0196. 30707656 10.1089/tmj.2018.0196

[50] Saad M, Chan S, Nguyen L, Srivastava S, Appireddy R. Patient perceptions of the benefits and barriers of virtual postnatal care: a qualitative study. BMC Pregnancy Childbirth [Internet] 2021;21(1):543. doi: 10.1186/s12884-021-03999-9 34364367 PMC8346781

[51] LaBelle B, Franklyn AM, Nguyen VPKH, Anderson KE, Eibl JK, Marsh DC. Characterizing the Use of Telepsychiatry for Patients with Opioid Use Disorder and Cooccurring Mental Health Disorders in Ontario, Canada. Int J Telemed Appl [Internet]. 2018;2018:e7937610. Available from: https://www.hindawi.com/journals/ijta/2018/7937610/. 10.1155/2018/7937610 PMC5828243 29610570

[52] Wilkinson S, Borycki E, Kushniruk A. Best practices for EHR implementation: A BC First Nations community’s experience. Healthc Manage Forum [Internet]. 2020;33(1):39–46. doi: 10.1177/0840470419860863 31370716

[53] Schubert NJ, Backman PJ, Bhatla R, Corace KM. Telepsychiatry and patient–provider concordance. Can J Rural Med [Internet]. 2019;24(3):75. Available from: https://journals.lww.com/cjrm/fulltext/2019/24030/telepsychiatry_and_patient_provider_concordance.5.aspx. doi: 10.4103/CJRM.CJRM_9_18 31249155

[54] Navarro JM, Scheim AI, Bauer GR. The Preferences of Transgender and Nonbinary People for Virtual Health Care After the COVID-19 Pandemic in Canada: Cross-sectional Study. J Med Internet Res. 2022 Oct 26;24(10):e40989. doi: 10.2196/40989 36170497 PMC9611101

[55] Singh J, Lou A, Green M, Keely E, Greenaway M, Liddy C. Evaluation of an electronic consultation service for transgender care. BMC Fam Pract [Internet]. 2021;22(1):55. doi: 10.1186/s12875-021-01401-3 33743596 PMC7980551

[56] Joschko J, Liddy C, Moroz I, Reiche M, Crowe L, Afkham A, et al. Just a click away: exploring patients’ perspectives on receiving care through the Champlain BASETM eConsult service. Fam Pract [Internet]. 2018;35(1):93–8. doi: 10.1093/fampra/cmx073 28968806

[57] Toulany A, Kurdyak P, Stukel TA, Strauss R, Fu L, Guan J, et al. Sociodemographic Differences in Physician-Based Mental Health and Virtual Care Utilization and Uptake of Virtual Care Among Children and Adolescents During the COVID-19 Pandemic in Ontario, Canada: A Population-Based Study. Can J Psychiatry. 2023 Dec 28;68(12):904–15. doi: 10.1177/07067437231156254 36855797 PMC9982398

[58] Moroz I, Archibald D, Breton M, Cote-Boileau E, Crowe L, Horsley T, et al. Key factors for national spread and scale-up of an eConsult innovation. Health Res Policy Syst [Internet]. 2020;18(1):57. doi: 10.1186/s12961-020-00574-0 32493357 PMC7268606

[59] Hynie M, Jaimes A, Oda A, Rivest-Beauregard M, Perez Gonzalez L, Ives N, et al. Assessing Virtual Mental Health Access for Refugees during the COVID-19 Pandemic Using the Levesque Client-Centered Framework: What Have We Learned and How Will We Plan for the Future? Int J Environ Res Public Health. 2022 Apr 20;19(9):5001. doi: 10.3390/ijerph19095001 35564397 PMC9103707

[60] Rush KL, Seaton C, Li E, Oelke ND, Pesut B. Rural use of health service and telemedicine during COVID-19: The role of access and eHealth literacy. Health Informatics J. 2021 Apr 27;27(2):146045822110200. doi: 10.1177/14604582211020064 34041936

[61] Lam J, Ahmad K, Gin K, Chow CM. Deliver Cardiac Virtual Care: A Primer for Cardiovascular Professionals in Canada. CJC Open [Internet]. 2022;4(2):148–157. Available from: https://www.cjcopen.ca/article/S2589-790X(21)00270-5/fulltext. doi: 10.1016/j.cjco.2021.10.001 34661090 PMC8502077

[62] Ennis M, Wahl K, Jeong D, Knight K, Renner R, Munro S, et al. The perspective of Canadian health care professionals on abortion service during the COVID-19 pandemic. Fam Pract [Internet]. 2021;38(Supplement_1):i30–6. doi: 10.1093/fampra/cmab083 34448482 PMC8414916

[63] Paprica PA, Sutherland E, Smith A, Brudno M, Cartagena RG, Crichlow M, et al. Essential Requirements for Establishing and Operating Data Trusts. Int J Popul Data Sci. 2020 Aug 24;5(1). 10.23889/ijpds.v5i1.1353 PMC7894384 33644412

[64] Zhou A, Osman A, Flores G, Srikrishnaraj D, Mohanty J, Al Bader R, et al. Critical Illness in Migrant Workers in the Windsor-Essex Region: A Descriptive Analysis. Int J Environ Res Public Health. 2023 Aug 16;20(16):6587. doi: 10.3390/ijerph20166587 37623172 PMC10454922

[65] Isenberg SR, Bonares M, Kurahashi AM, Algu K, Mahtani R. Race and birth country are associated with discharge location from hospital: A retrospective cohort study of demographic differences for patients receiving inpatient palliative care. EClinicalMedicine. 2022 Mar;45:101303. doi: 10.1016/j.eclinm.2022.101303 35243270 PMC8860918

[66] Heald FA, Marzolini S, Colella TJF, Oh P, Nijhawan R, Grace SL. Women’s outcomes following mixed-sex, women-only, and home-based cardiac rehabilitation participation and comparison by sex. BMC Womens Health [Internet]. 2021;21(1):413. doi: 10.1186/s12905-021-01553-5 34911506 PMC8672337

[67] Kundu A, Fu R, Grace D, Logie C, Abramovich A, Baskerville B, et al. Correlates of past year suicidal thoughts among sexual and gender minority young adults: A machine learning analysis. J Psychiatr Res. 2022 Aug;152:269–77. doi: 10.1016/j.jpsychires.2022.06.013 35759979

[68] Taylor J, Kukutai T. Indigenous Data Sovereignty [Internet]. ANU Press; 2023. Available from: https://press.anu.edu.au/publications/series/caepr/indigenous-data-sovereignty. 10.1126/science.adl4664 38033063

[69] Chedid RA, Terrell RM, Phillips KP. Best practices for online Canadian prenatal health promotion: A public health approach. Women Birth. 2018 Aug;31(4):e223–31. doi: 10.1016/j.wombi.2017.10.005 29113753

[70] Boelen C, Blouin D, Gibbs T, Woollard R. Accrediting Excellence for a Medical School’s Impact on Population Health. Educ Health [Internet]. 2019;32(1):41. Available from: https://journals.lww.com/EDHE/Fulltext/2019/32010/Accrediting_Excellence_for_a_Medical_School_s.8.aspx. 10.4103/efh.EfH_204_19 31512592

[71] Polisena J, Garritty C, Kamel C, Stevens A, Abou-Setta AM. Rapid review programs to support health care and policy decision making: a descriptive analysis of processes and methods. Syst Rev [Internet]. 2015;4(1):26. doi: 10.1186/s13643-015-0022-6 25874967 PMC4407715

[72] Saeed SA, Masters RM. Disparities in Health Care and the Digital Divide. Curr Psychiatry Rep [Internet]. 2021;23(9):61. doi: 10.1007/s11920-021-01274-4 34297202 PMC8300069

[73] Boelen C, Woollard R. Social accountability: The extra leap to excellence for educational institutions. Med Teach [Internet]. 2011;33(8):614–9. doi: 10.3109/0142159X.2011.590248 21774646

[74] Khangura S, Konnyu K, Cushman R, Grimshaw J, Moher D. Evidence summaries: the evolution of a rapid review approach. Syst Rev [Internet]. 2012;1(1):10. doi: 10.1186/2046-4053-1-10 22587960 PMC3351736

[75] Ganann R, Ciliska D, Thomas H. Expediting systematic reviews: methods and implications of rapid reviews. Implement Sci [Internet]. 2010;5(1):56. doi: 10.1186/1748-5908-5-56 20642853 PMC2914085

[76] Wilson MG, Oliver S, Melendez-Torres GJ, Lavis JN, Waddell K, Dickson K. Paper 3: Selecting rapid review methods for complex questions related to health policy and system issues. Syst Rev [Internet]. 2021;10(1):286. doi: 10.1186/s13643-021-01834-y 34717777 PMC8556903

[77] Ferrazzi P, Krupa T. Remoteness and its impact on the potential for mental health initiatives in criminal courts in Nunavut, Canada. Int J Circumpolar Health. 2018 Jan;77(1):1541700. doi: 10.1080/22423982.2018.1541700 30384817 PMC6225482

[78] Brownell M, Enns JE, Hanlon-Dearman A, Chateau D, Phillips-Beck W, Singal D, et al. Health, Social, Education, and Justice Outcomes of Manitoba First Nations Children Diagnosed with Fetal Alcohol Spectrum Disorder: A Population-Based Cohort Study of Linked Administrative Data. Can J Psychiatry. 2019 Sep 30;64(9):611–20. doi: 10.1177/0706743718816064 30595040 PMC6699031

[79] Baliunas D, Zawertailo L, Voci S, Gatov E, Bondy SJ, Fu L, et al. Variability in patient sociodemographics, clinical characteristics, and healthcare service utilization among 107,302 treatment seeking smokers in Ontario: A cross-sectional comparison. PLoS ONE. 2020 Jul 10;15(7):e0235709. doi: 10.1371/journal.pone.0235709 32650339 PMC7351500

[80] Kueper JK, Rayner J, Zwarenstein M, Lizotte D. Describing a complex primary health care population to support future decision support initiatives. Int J Popul Data Sci. 2022 Oct 24;7(1). doi: 10.23889/ijpds.v7i1.1756 37670733 PMC10476014

[81] Government of Canada. Canada Health Act [Internet]. Ottawa; 1985 [cited 2024 Jul 2]. Available from: https://laws-lois.justice.gc.ca/PDF/C-6.pdf.

[82] Khangura S, Polisena J, Clifford TJ, Farrah K, Kamel C. RAPID REVIEW: AN EMERGING APPROACH TO EVIDENCE SYNTHESIS IN HEALTH TECHNOLOGY ASSESSMENT. Int J Technol Assess Health Care [Internet]. 2014;30(1):20–27. Available from: https://www.cambridge.org/core/journals/international-journal-of-technology-assessment-in-health-care/article/rapid-review-an-emerging-approach-to-evidence-synthesis-in-health-technology-assessment/11B54AE04F21C14C20FB4F1730CDD709. doi: 10.1017/S0266462313000664 24451157

[83] Brey P, Søraker JH. Philosophy of Computing and Information Technology. In: Meijers A, editor. Philosophy of Technology and Engineering Sciences [Internet]. Amsterdam: North-Holland; 2009. p. 1341–407. (Handbook of the Philosophy of Science). Available from: https://www.sciencedirect.com/science/article/pii/B9780444516671500513.

