# Supplementary material for: Artificial intelligence and social accountability in the Canadian health care landscape: A rapid literature review
Source: PLOS Digit Health. 2024 Sep 12;3(9):e0000597. doi: 10.1371/journal.pdig.0000597 (PMC11392241; doi:10.1371/journal.pdig.0000597)
Supplement: S1 File — (PDF) [file pdig.0000597.s001.pdf]

Authors	Year	Title	Journal	Location	Study Type	Design/Methodology	Population	Objective	Artificial Intelligence Technology	Conclusions	Domain (education, service delivery, research)	micro, meso, macro
Jose M Navarro, Ayden I Scheim, Greta R Bauer	2022	The Preferences of Transgender and Nonbinary People for Virtual Health Care After the COVID-19 Pandemic in Canada: Cross-sectional Study	Journal of medical internet research	Canada	mixed methods	Chi-square tests were used to compare postpandemic preferences for virtual versus in-person care across sociodemographic, health, and social characteristics. Participants provided open-text responses explaining their preferences, which were used to contextualize quantitative findings.	TransPULSE Canada COVID survey collected national data on thehealth, economic, and social impacts of the COVID-19pandemic on TNB people in Canada	This study aimed to identify the sociodemographic, health, and social factors associated with postpandemic virtualcare preferences in TNB communities	virtual care	TNB people may have differential interest in virtual care based on factors including age, chronic and mental health conditions, and gender-unsupportive home environments. Future research examining virtual care preferences would benefit from mixed methods intersectional approaches across these factors, to explore complexity in the barriers and facilitators of virtual care access and quality. These observed differences support flexibility with options to choose between in-person and virtual healthcare to meet TNB patients'specific health needs.	service delivery	meso
Sara Bhatti, Simone Dahrouge, Laura Muldoon, Jennifer Rayner	2022	Using the quadruple aim to understand the impact of virtual delivery of care within Ontario community health centres: a qualitative study	BJGP Open	Ontario	qualitative	Thematic analysis of qualitative interviews. Semi-structured phone interviews were conducted in the autumn of 2020. Interviews were recorded, transcribed verbatim, and analysed thematically using the quadruple aim framework.	Ontario community health centres (CHCs) providers and patients	To understand the impact of virtual care on healthcare system performance within the context of Ontario community health centres (CHCs)	virtual care	The study described positive and negative impacts on patient care, population health, health system costs, and provider experience. These results will be useful for primary care organisations in post-pandemic planning; however, future research is needed for a deeper exploration of the impact on quality of care specifically for more complex health concerns.	service delivery	meso
Alene Toulany, Paul Kurdyak, Therese A. Stukel, Rachel Strauss, Longdi Fu, Jun Guan, Lisa Fiksenbaum, Eyal Cohen, Astrid Guttmann, Simone Vigod, Maria Chiu, Charlotte Moore Hepburn, Kimberly Moran, William Gardner, Mario Cappelli, Purnima Sundar, and Natasha Saunders	2023	Sociodemographic Differences in Physician-Based Mental Health and Virtual Care Utilization and Uptake of Virtual Care Among Children and Adolescents During the COVID-19 Pandemic in Ontario, Canada:A Population-Based Study	The Canadian Journal of Psychiatry	Ontario	quantitativ e	This population-based repeated cross-sectional study of children and adolescents (3–17 years;N=2.5 million)used linked health and demographic administrative data in Ontario, Canada (2017–2021). Multivariable Poisson regressions with generalized estimating equations compared rates of outpatient physician-based mental healthcare use during thefirst yearof the COVID-19 pandemic with expected rates based on pre-COVID patterns. Analyses were conducted by socioeconomicstatus (material deprivation quintiles of the Ontario Marginalization index), urban/rural region of residence, and immigrationstatus.	children and adolescents ages 3–17 years living in Ontarioand eligible for provincial health insurance	We sought to evaluate the relationship between social determinants of health and physician-based mental health-care utilization and virtual care use among children and adolescents in Ontario, Canada, during the COVID-19 pandemic.	virtual care	During the first year of the pandemic, pediatric physician-based mental healthcare utilization was higher among immigrants and lower than expected among those with lower socioeconomic status. Refugees had the lowest use of virtual care. Further work is needed to understand whether these differences reflect issues in access to care or the need to help inform ongoing pandemic recovery planning.	service delivery	meso
Simone Shahid, Sophie Hogeveen, Philina Sky, Shivani Chandra, Suman Budhwani, Ryande Silva, R.Sacha Bhatia, Emily Seto and James Shaw	2023	Health equity related challenges and experiences during the rapid implementation of virtual care during COVID-19: a multiple case study	International Journal for Equity in Health	Canada	qualitative	This study followed an exploratory, multiple case study approach drawing on the methodological guidance of Yin [28]. We completed exploratory case studies of health and social service organizations that rapidly implemented virtual care during the initial onset of the COVID-19 pandemic in Canada across multiple care settings in Ontario. We recruited participants through a single study con-tact (gatekeeper) at each participating organization. We conducted semi-structured qualitative inter-views with participants from all four case studies during the second phase (July 2020 to March 2021) of the COVID-19 Pandemic in Canada. We engaged in rapid analytic methods in order to analyze the interview data.		The objective of this paper is to1)document the challenges in considering health equity during the rapid implementation of virtual care in Ontario, Canada during COVID-19,2)document the strategies implemented to mitigate challenges, and3)explore how access was hindered or achieved based on the results.	virtual care	The inequitable distribution of health care resources arising from persistent colonial systems and practices have far-ranging consequences, and these include their impact on inequitable access to virtual care.	service delivery	meso
Jacqueline K. Kueper, Jennifer Rayner, Merrick Zwarenstein, Daniel J. Lizotte	2022	Describing a complex primary health care population in a learning health system to support future decision support and artificial intelligence initiatives	BJGP Open	Ontario	qualitative	We use electronic health record data from 2009-2019 to describe sociodemographic, clinical, and health care use characteristics of adult primary care clients served by the Alliance for Healthier Communities. In addition to simple summary statistics, we apply unsupervised leaning techniques to explore patterns of common condition co-occurrence, care provider teams, and care frequency	ongoing primary care clients served by the Alliance. de-identified extract of the centralized, structured EHR database from all CHCs.	Our objective is to summarize sociodemographic, clinical, and health care use characteristics of this population	AI	This population-level overview of clients served by the Alliance provides a foundation for future LHS initiatives. In addition to substantive findings, we demonstrate the use of methods from statistics and artificial intelligence to describe a complex primary care population. We discuss implications for future initiatives, including development of decision 47 support tools.	research	macro
Karine Latulippe;	2020	Co-Design to Support the Development of Inclusive eHealth	Journal of	Quebec	qualitative	This study is part of a larger project titled, "Better	All QADA project co-	The objective of this paper	virtual care	The use of co-design involving participants at risk of SHIs	service delivery	micro
Kathy L Rush, Cherisse Seaton, Eric Li, Nelly D Oelke, Barbara Pesut	2021	Rural use of health service and telemedicine during COVID-19: The role of access and eHealth literacy	Health Informatics Journal	rural British Columbi a	qualitative	This study employed a cross-sectional online survey from May 29 to July 8, 2020. Measures: Demographic characteristics of participants, Health care service use, Health service access and frequency of use, Telemedicine use and satisfaction, eHealth literacy, Telemedicine experiences and suggestions	Participation was open to all rural British Columbia community members community members; however, recruitment efforts targeted those communities where medical services were provided through one of the provincial health regions.	Research questions1.What proportion of rural community participants accessed health services, mental health services, and telemedicine during COVID-19?2.How satisfied are rural community members with telemedicine?3.How is telemedicine satisfaction related to eHealth literacy?4.What suggestions do participants have for improving telemedicine in rural areas?	telehealth	In conclusion, during the COVID-19 pandemic, two thirds of rural residents in a Canadian western province self-reported having a health care need, the majority had access to telemedicine, and just under half had used it. Compared to pre-COVID-19, self-reported telemedicine use had increased during COVID-19 as had use of mental health online programs and virtual services. E-Health literacy was positively associated with satisfaction with telemedicine. Yet, rural citizens also experienced challenges with telemedicine access due to unreliable/unstable internet and found the service impersonal. The lack of digital infrastructure is an ongoing concern for rural communities if they are to experience health care equity comparable to their urban counterparts. If telemedicine is to be incorporated into practice during and following COVID-19, it is important to ensure that rural residents have the support needed to engage with this form of remote care.	research	meso

Madeleine Ennis, Kate Wahl, Dahn Jeong, Kira Knight, Regina Renner, Sarah Munro, Sheila Dunn, Edith Guilbert and Wendy V Norman	2021	The perspective of Canadian health care professionals on abortion service during the COVID-19 pandemic	Family Practice	Canada	mixed methods	We adapted the Standards for Reporting Qualitative Research Checklist for a mixed-methods approach (22). We conducted this second survey to further understand and quantify factors identified in the first survey through a refined set of questions and in response to a request from federal regulatory stakeholders. codebook thematic analysis of open-ended responses following an inductive approach	We conducted an exploratory sequential mixed methods study that involved the collection and analysis of qualitative data from our 2019 Canadian Abortion Provider Survey (21) which, in turn, in-formed the development of a second primarily quantitative survey.	We sought to characterize the experiences of abortion health care professionals in Canada during the COVID-19 pandemic and the impact of the pandemic response on abortion services	telehealth	Canadian health care professionals report their facilities deemed abortion an essential service. Provinces and territories, except Quebec, described a robust pandemic transition to telemedicine to ensure access to services	research	macro
Michaela Hynie, Annie Jaimes, Anna Oda, Marjolaine Rivest-Beauregard, Laura Perez Gonzalez,Nicole Ives, Farah Ahmad, Ben C. H. Kuo, Neil Arya, Nimo Bokore and Kwame McKenzie	2022	Assessing Virtual Mental Health Access for Refugees duringthe COVID-19 Pandemic Using the Levesque Client-CenteredFramework: What Have We Learned and How Will We Plan forthe Future?	International Journal of Environmental Research and Public Health	Alberta, British Columbia, Ontario, and Quebec	qualitative	Questions regarding providers' perceptions of challenges in the delivery of VMH care andaccess to resources and training made up the mixed-methods part of the study. This paper reports on the qualitative assessment of access to VMH services using data from interviews with community leaders, health and mental health providers, managers and newcomer clients, and front-line providers. Focus groups were planned with providers,but aside from 6 small group interviews, individual interviews were utilized instead due to recruitment challenges.	11 providers and policy makers working with refugee newcomers in the four provinces,the second of 11 newcomers from the Afghan, Congolese, Eritrean, Ethiopian, Iranian, and Syrian communities in these same provinces	this project aimed to better understand the accessibility of VMH careduring the COVID-19 pandemic from the perspective of both refugee newcomer clientsand providers offering or referring to VMH services, to support access to more equitable,effective, and appropriate VMH services for refugee newcomers across Canada	telehealth	As we transition back to more in-person care, almost all providers noted a desire to retain some elements of VMH care in the future. Thus, although the circumstances of delivering mental health services during the pandemic were unique, they also offered opportunities to learn more about whether, for whom, when, and how virtual mental health care increases access to services. VMH services rapidly expanded in the COVID-19 context and have the potential to bridge gaps between refugee mental health care needsand available services. However, most virtual health initiatives are not sustained becauseof a lack of research on user needs, goals, and perceptions [17,18], and fail to addressaccessibility barriers for disadvantaged patients [42]. This study identified a number ofcharacteristics of VMH services that could interact with clients' abilities, to either limit orenhance access to needed mental health care. Importantly, virtual modalities differed inaccessibility as a function of the services offered, client needs, abilities, and preferences,and the resources available, reinforcing the importance of flexibility and choice in VMHservices to reflect the diversity of refugee client circumstances and needs.	service delivery	macro
Jessica Cao; Tina Felfeli; Rebecca Merritt; Michael H. Brent	2022	Sociodemographics Associated With Risk of Diabetic Retinopathy Detected by Tele-Ophthalmology: 5-Year Results of the TorontoTele-Retinal Screening Program	Canadian Journal of Diabetes	Toronto	quantitative	A prospective cohort study was conducted on adults with diabetes type 1 or type 2 enrolled inthe Toronto Tele-Retinal Screening Program between September 2013 and March 2019. Descriptive statistics were used to report sociodemographic data and screening outcomes.	All participants enrolled in the Toronto Tele-Retinal Screening Program between September 2013 and March2019. he study population consisted of adults 18 years of age, diagnosed with type 1 or type 2 diabetes, and who had not received an eye examination in the previous 12 months	we aim to characterize thesociodemographic makeup of participants enrolled in the TTRSPand their associations with screening outcomes	telehealth	In conclusion, the TTRSP has been effective in identifying DR inone-quarter of all screens in an inner-city setting for over 5 years.Data show that participants in a lower income bracket have signif-icantly increased odds of screening positive for DR. Sociodemographic analysis has revealed an urban, culturally diverse population with low SES. A substantial minority of participants lived below the poverty line, had a low level of education or did not have universal health coverage. Further scaling-up and expansion of tele-ophthalmology programs will provide more robust data to address DR screening needs in vulnerable communities. Application of tele-ophthalmologic DR screening in an international setting can be instrumental in reducing the worldwide burden of DR.	service delivery	micro
Paige Dean, Maureen O'Donnell, Lenny Zhou, Erik D. Skarsgard	2019	Improving value and access to specialty medical care for families: a pediatric surgery telehealth program	Canadian journal of surgery	rural British Columbia	qualitative	We analyzed pediatric surgery consultation Wait 1 (the time from referral to consultation) data before and after telehealth implementation to determine whether integration of a telehealth alternative to inperson consultation had a beneficial impact on overall accessAppointments were initially scheduled at 45minute intervals, with 15 minutes between appointments; however, later in the experience, some appointments were scheduled at 30minute intervals.At the end of the encounter, both the parent and patient (if appropriate) provided feedback via a questionnaire, which included an option for narrative comments . Satisfaction of the provider (telehealth provider and telehealth support staff) was also assessed by way of a questionnaire	Follow up for patients from remote communities who had undergone surgical procedures at BC Children's Hospital. However, as experience increased, consultation was offered increasingly for new patient referrals.	The objective of this pilot project was to explore the patient/provider experience with telehealth in the context of pediatric surgery and to evaluate the impact of a telehealth alternative on overall access to pediatric surgical consultation.	telehealth	Our findings suggest that virtual care provided through a pediatric surgical telehealth clinic has high value for fam-ilies from remote communities in terms of encounter quality and travel cost avoidance. Virtual pediatric surgical care is an effective complement to inperson ambulatory care from the provider perspective, and it maximizes capacity of hospitalbased clinics for those who require access to it. The role of telehealth in delivery of health care services is likely to increase as enabling information and communication technology becomes more widely adopted. Given the demands for access to subspecialty children's surgical consultation, investment in infrastructure to support telehealth benefits families who are geographically remote from the referral centre. This technology also has the potential to benefit delivery of children's surgical services overall by increasing system capacity. Pediatric surgeons should be open to the possibility of providing telehealth consultation but must be attentive to its limitations.	service delivery	micro

P. Alison Paprica, Eric Sutherland, Andrea Smith, Michael Brudno, Rosario G. Cartagena, Monique Crichlow, Brian K. Courtney, Chris Loken, Kimberlyn M. McGrail, Alex Ryan, Michael J. Schull, Adrian Thorogood, CarlVirtanen, and Kathleen Yang	2020	Essential requirements for establishing and operating data trusts: practical guidance co-developed by representatives from fifteen canadian organizations and initiatives	International Journal of Population Data Science	Canada	qualitative	Each participating organization/initiative was asked toprovide a written summary of its activities which wascirculated in advance of the meeting. We held a six-hour in-person meeting in Toronto on December 3, 2019which comprised brief (~5 minute) presentations about eachorganization/initiative, followed by a series of facilitateddiscussions. The meeting utilized a minimum specificationsrequirements ("min specs") approach to identify the essentialelements and key characteristics of data trusts. Liveinternet polling was used to capture individual suggestionsand key points from the group discussions. Preliminary minspecs requirements were presented during the meeting. Themin specs (Box 1) were refined as authors contributed theirsuggestions to a rapid literature review (performed usinga snowball search method) and worked collaboratively oniterations of this manuscript	In total 19 people representing 15 organizations and datainfrastructure initiatives participated. Most participants workat publicly funded organizations focused on health dataand/or data associated with publicly funded services. Severalparticipants were involved in more than one initiativeor organization, including some commercial organizations	Our aim was to combineour first-hand experience of Canadian data infrastructure witha synthesis of concepts from related literature to establish acommon understanding of the essential requirements for datatrusts, irrespective of the form that a data trust may take.	health informatics/AI	Based on our experience with data infrastructure in Canada, we identified a relatively small number (12) of min specs for establishing and operating data trusts which should be practical to implement. The mechanism of a Capability Exchange combined with min specs facilitation was effective for identifying essential requirements for data trusts. This feature paper is just a start; continued joint work with members of the public, representatives from commercial organizations and from other Canadian and international organizations involved in data infrastructure is recommendedon this evolving topic.	service delivery	macro
Dolly BaliunasI, Laurie Zawertailo, Sabrina Voci, Evgenia Gatov, Susan J. Bondy ,Long di Fu ,Peter L. Selby	2020	Variability in patient sociodemographics, clinical characteristics, and healthcare service utilization among 107,302 treatment seeking smokers in Ontario: A cross-sectional comparison	PLOS ONE	Ontario	quantitativ e	descriptive cross-sectional study	all individuals who sought smoking cessation treatment via the STOP program in Ontario, Canada	our objective was to identify and characterize any variation in the population of treatment seeking smokers reached by the different treatment models offered by STOP.	health informatics	Practical guidance for the establishment and operation of data trusts was articulated in the form of 12 min specs requirements. The 12 min specs are a starting point. Future work to refine and strengthen them with members of the public, companies, and additional research data stakeholders from within and outside of Canada, is recommended.	service delivery	meso
Winta Ghidei, Stephanie Montesanti, Lana Wells and Peter H. Silverstone	2022	Perspectives on delivering safe and equitable trauma-focused intimate partner violence interventions via virtual means: A qualitative study during COVID-19 pandemic.	BMC public health	Alberta	quantitativ e	semi-structured interviews; The interviews focused on the perspectives and experiences of the providers as an indirect source of information about virtual delivery of IPV interventions for a diverse range of individuals affected by IPV. Interview transcripts were analyzed using inductive thematic analysis	24 service providers within the anti-violence sector in Alberta, Canada	This study aimed to qualitatively describe the challenges experienced by service providers with delivering virtually delivered IPV services that are safe, equitable, and accessible for their diverse clients during the COVID-19 pandemic	virtual care	The findings from this qualitative research identified key determining factors for delivering safe, equitable, and accessible virtually delivered intervention for a diverse range of populations. To ensure virtual interventions are safe and equitable it is necessary for service providers to acknowledge and attend to underlying	service delivery	meso
Anasua Kundu, Rui Fud, Daniel Grace, Carmen Logie, Alex Abramovich, Bruce Baskerville, Christina Yager, Robert Schwartz, Nicholas Mitsakakis, Lynn Planinac, Michael Chaiton	2022	Correlates of past year suicidal thoughts among sexual and gender minority young adults: A machine learning analysis.	Journal of psychiatric research	Ontario and Quebec	qualitative	Cross-sectional online survey; random forest model to predict risk of having past year suicidal thoughts	LGBTQI2S +participants aged 16–29 years living in two Canadian provinces (Ontario, Quebec).	We aimed to examine the relative importance and effects of intersectional factors and strong interactions associated with the risk of suicidal thoughts among Canadian lesbian, gay, bisexual, transgender, queer, ques-tioning, intersex and Two Spirit (LGBTQI2S+) young adults.	machine learning analysis	The increase in the risk of suicidal thoughts for those having mental health challenges or facing minority stressors is more pronounced in those living in urban areas or being unemployed than those living in rural areas or being employed	service delivery	macro
Megan Saad, Sophy Chan, Lisa Nguyen, Siddhartha Srivastava, and Ramana Appireddy	2021	Patient perceptions of the benefits and barriers of virtual postnatal care: a qualitative study.	BMC pregnancy and childbirth	Kingston , Ontario	qualitative	Semi-structured interviews; interviews were 20–25min in length and recorded through an audio recorder. Thematic analysis was conducted in order to derive the major themes explored in this study	15 patients attending the Kingston Health Sciences Centre	The objective of this study is to understand the perceptions of new mothers using virtual care via video conferencing to gain insight into the benefits and barriers of virtual care for obstetric patients	virtual care	Virtual care is a useful modality that could improve compliance to obstetric care. Further research and clinical endeavours should examine how social factors and determinants intersect to determine how they underpin patient perceptions of virtual and in-person care.	service delivery	micro
Isabella Moroz, Douglas Archibald, Mylaine Breton, Elizabeth Cote-Boileau, Lois Crowe, Tanya Horsley, Lirjie Hyseni, Gina Johar, Erin Keely, Katharina Kovacs Burns, Craig Kuziemsky, Jim Laplante, Ariana Mihan, Luis Oppenheimer, Don Sturge, Delphine S. Tuot, and Clare Liddy	2020	Key factors for national spread and scale-up of an eConsult innovation	Health research policy and systems	Ontario	qualitative	Constant comparative thematic analysis. Proceedings were recorded, transcribed and underwent qualitative analysis using the Framework for Applied Policy Research	Sixty-four participants from a National eConsult Forum meeting in Ottawa, Canada. Representing provincial and territorial governments, national organisations, healthcare providers,researchers and patients.	we aimed to identify the key factors involved in the spread and scale-up of a successful regional eConsult modelacross Canada.	eConsult	Efforts to promote innovation in healthcare are more likely to succeed if they are based on anunderstanding of the forces that drive the spread and scale-up of innovation. Further research is needed todevelop and strengthen the conceptual and applied foundations of the spread and scale-up of healthcareinnovations, especially in the context of emergent learning health systems across Canada and beyond	service delivery	meso

Susi Wilkinson, Elizabeth Borycki, Andre Kushniruk	2020	Best practices for EHR implementation: A BC First Nations community's experience	Healthcare management forum	British Columbia FN	mixed methods	This study used qualitative and quantitative methods. Retrospective, mixed methods study was conducted	First Nations Health Centre in BC, Canada. he FN community's territory isadjacent to two cities with populations 33,000 and 9,000, andmost of the FN community's 1,000 members live within 4 kmof the larger city. The FN health team consists of eight part-time staff and one health director	The objective of this study was to validate the EHRIF in the implementation of a community EHR in a BC First Nations community. The study asked the following questions: Which success factors were present and missing in the pre-implementation and implementation phases of the EHR implementation? How do the implementation team members rate the importance of the success factors in their contribution to the EHR implementation?	EMR	This study found the EHRIF was valid in the context of anEMR implementation in a FN community. resolution of privacy and confidentiality issues was criticallyimportant for the EMR implementation to proceed and that collaboration with a local physician champion, and provincial and federal governments, combined with demonstratedcommitment from all parties was necessary for the EMRimplementation to occur.	service delivery	micro
Brittanie LaBelle, Alexandra M. Franklyn, Vicky PKH Nguyen, Kathleen E. Anderson, Joseph K. Eibl, and David C. Marsh	2018	Characterizing the Use of Telepsychiatry for Patients with Opioid Use Disorder and Cooccurring Mental Health Disorders in Ontario, Canada	International Journal of Telemedicine and Applications	Northern /Southern Ontario	quantitative	A retrospective cohort study was conducted using an administrative database forpatients who received psychiatric services via telemedicine between 2008 and 2014 and who also had OUD	9,077 patients with concurrent opioid use and other mental health disorders who had received psychiatric services via telemedicinefrom 2008 to 2014; 7,109 (78.3%) patients lived in Southern Ontario and 1,968 (21.7%) in Northern Ontario	We characterize the usage of telemedicine to deliver psychiatric services to patients with opioid use disorder (OUD) in Ontario, as well as traits of treatment-seeking patients with opioid dependence and concurrent psychiatric disorders.	telehealth	Telemedicine is increasingly being utilized throughout Ontario for delivering mental health treatment. There is an opportunity to increase access to psychiatric services for patients with opioid dependence and concurrent psychiatric disorders through the use of the telemedicine	service delivery	meso
Marni Brownell, Jennifer E. Enns, Ana Hanlon-Dearman, Dan Chateau, Wanda Phillips-Beck, Deepa Singal, Leonard MacWilliam, Sally Longstaffe, Ab Chudley, Brenda Elias, and Noralou Roos	2019	Health, Social, Education, and Justice Outcomes of Manitoba First Nations Children Diagnosed with Fetal Alcohol Spectrum Disorder: A Population-Based Cohort Study of Linked Administrative Data	Canadian Journal of Psychiatry	Manitoba FN	quantitative	Retrospective cohort study, health and social services, education, and justice data were linked with clinicalrecords on First Nations (FN) individuals aged 1 to 25 and diagnosed with FASD between 1999 and 2010 (n¼743). Wecompared the FN FASD group to non-FN individuals with FASD (non-FN FASD;n¼315) and to First Nations individuals(matched on age, sex, and income) not diagnosed with FASD (FN non-FASD;n¼2229). Rates and relative risks (RRs) werecalculated using generalized linear models	1058 children and youth in Manitoba to healthand social service use, education, and justice records held inthe Manitoba Population Research Data Repository at theManitoba Centre for Health Policy (MCHP). ompared health, social,and education outcomes for First Nations children and youthdiagnosed with FASD to non–First Nations children andyouth with FASD and First Nations children and youth with-out FASD	To examine health services, social services, education, and justice system outcomes among First Nations childrenand youth with fetal alcohol spectrum disorder (FASD)	health informatics	Young people with FASD are at risk for poor health, education, and social outcomes, but First Nations youngpeople with FASD face comparably higher risks, particularly with child welfare and justice system involvement. The study emphasizes a critical need for appropriate resources for First Nations children with FASD	service delivery	meso
Jeffrey Lam, Kamran Ahmad, Kenneth Gin and Chi-Ming Chow	2022	Deliver Cardiac Virtual Care: A Primer for Cardiovascular Professionals in Canada	CJC Open	Canada	qualitative		Canada	This review article aims to provide a primer on virtual care for cardiovascular professionals in Canada	virtual care	These initial experiences of CVC need to be studied carefully in terms of cardiovascular outcomes as well as the processes of care delivery and their quality. There have been many successes with CVC delivery, but many initial concerns have been raised with respect to patient privacy, access to technology, language discrepancies, and provider remuneration. Cardiac diagnostic testing and therapeutic procedures remain mostly in the realm of in-person care. Nevertheless, the groundwork has been laid for virtual care to remain as a key modality for cardiovascular health care delivery in Canada. Ongoing study will be required to continue to refine and improve it and to understand its role alongside traditional in-person care	service delivery	macro
Sheldon Cheskes, Shelley L. McLeod, Michael Nolan, Paul Snobelen, Christian Vaillancourt, Steven C. Brooks, Katie N. Dainty, Timothy C. Y. Chan, Ian R. Drennan	2020	Improving Access to Automated External Defibrillators in Rural and Remote Settings: A Drone Delivery Feasibility Study	Journal of the Amerian Heart Association	rural Southern Ontario	quantitative	Conducted 6 simulations. n the first 2 simu-lations, the drone and ambulance were dispatched from the same paramedic base. In simulations 3 and 4, the drone and ambulance were dispatched from separate paramedic bases; and in simulations 5 and 6, the drone was dispatched from an optimized location. During each simulation, a "mock" call was placed to 911 and a single AED drone and an ambulance were simultaneously dispatched to a predetermined destination. On scene, trained first responders retrieved the AED from the drone and initiated resuscitative efforts on a mannequin until paramedics arrived	2 rural communities in southern Ontario, Canada	Our primary objective was to ex-amine the feasibility of a novel AED drone delivery method for rural and remote sudden cardiac arrest. A secondary objective was to compare response times between AED drone delivery and ambulance to mock sudden cardiac arrest resuscitations.	robotics	This study suggests AED drone delivery is feasible, with the potential for improvements in response time during simulated sudden cardiac arrest scenarios. Further research is required to determine the appropriate system configuration for AED drone delivery in an integrated emergency medical service system as well as optimal strategies to simplify bystander application of a drone-delivered AED	service delivery	micro

Tyler Marshall, Dylan Viste, Stephanie Jones, Julia Kim, Amanda Lee, Farah Jafri, Oona Krieg and S. Monty Ghosh	2023	Beliefs, attitudes and experiences of virtual overdose monitoring services from the perspectives of people who use substances in Canada: a qualitative study	Harm Reduction Journal	Canada	qualitative	A qualitative study was conducted that explored the per-ceptions and beliefs around VOMS from the perspectives of PWUS in Canada. Between February and March 2022, one-on-one telephone interviews were conducted with PWUS who have experience with VOMS (i.e., either as a client, volunteer or peer operator) over 18 years of age across Canada. It should be noted that many of the peer operators also used the VOMS regularly as a client when not on shift. Grounded theory methodology was used to guide the methods and analysis. Grounded theory was the most appropriate methodology for addressing our research question as it is commonly used to develop rich hypotheses and theories around complex phenom-ena involving the evaluation of public health programs	Canadian residents ≥18 years of age at the time of consent; Reported active use of unregulated substances (within the last seven days); Able to communicate effectively in English and pro-vide informed verbal consent; PWUS who worked in harm reduction or for VOMS were eligible.	The objective of this study was to explore the percep-tions and beliefs around VOMS from the perspectives of PWUS in Canada. This research will be used to develop hypotheses that will inform future research and service design	telehealth	VOMS may be useful harm reduction interventions pro-viding an adjunctive option for substance use monitoring and emergency response for PWUS alone and individuals who may experience barriers to accessing in-person SCS (e.g., due to quarantine, transportation, stigma, disabil-ity). Our data, in the context with the best available evi-dence, suggests VOMS may be most applicable for PWUS who have reliable access to technology, use substances alone and cannot access an in-person SCS. A novel and important finding also included that VOMS may also be beneficial at providing peer support and facilitating refer-rals to health and social services. However, due to poten-tial limitations around emergency response times and lack of in-person monitoring, we suggest using in-person SCS for providing overdose monitoring and supervision when possible. Since most substance-related overdoses occur during solitary substance use and away from an SCS, VOMS may be an important public health interven-tion for a population at high risk of mortality. It remains unclear whether VOMS will be feasible or widely utilized among people who live in rural/remote geographic areas or among individuals who do not have reliable telephone, cellular service or Wi-Fi access [30]. Anxiety about confi-dentiality and fear of arrest likely poses barriers to using VOMS and potentially other harm reduction services.	service delivery	meso
Aneisha Collins-Fairclough, Prabjit Barn, AJ Hirsch-Allen, Karen Rideout, Erin M Shellington, Winnie Lo, Tony Lanier, Jim Johnson, Adam Butcher,Sian-Hoe Cheong, Carmen Rempel, Nardia Strydom, Pat G Camp and Christopher Carlsten	2023	Disparities in self-reported healthcare access for airways disease in British Columbia, Canada, during the COVID-19 pandemic. Insights from a survey co-developed with people living with asthma and chronic obstructive pulmonary disease.	Chronic Respiratory Disease	BC	mixed methods	We used a patient-oriented research approach to co-design a study that would be acceptable to people living with asthma and COPD and obtain information that could be of interest to patients as well as clinicians and health care decision makers. We used a cross-sectional survey design as described below to understand the distribution of patients' perspectives on their access tocare. The STROBE checklist was used for reporting this observational study	18 yearsof age or older, self-reported doctor-diagnosed asthma or COPD of any duration; currently residing in BC; and ability to communicate in written English	The primary study objectiveswere to determine from the patient perspective: 1) whether their access to healthcare for asthma and COPD was re-duced after May 2020 when outpatient services (that had briefly been suspended) were partially restored, 2) which specific healthcare services, were disrupted after May 2020, and 3) whether there was differential access to care for different population subgroups after May 2020. We also aimed to describe patient-reported use of asthma and COPD services delivered by telehealth and identify patient characteristics associated with the use of different tele-health modalities.	telehealth	We sought to understand, from people living with asthmaand COPD in BC, how their access to asthma and COPD care was impacted after services were partially restored during the COVID-19 pandemic. We found that during this time, patients self-reported lower access to care, which was associated with low self-assessed financial ability, and that specialty care services were seen as most disrupted. It is important to reassess whether this disparity still exists post-pandemic. If it does, consideration of patient characteristics in healthcare quality improvement, and addressing the pathways by which disparities arise, could bepart of a health equity approach to restoring routine healthcare services for asthma and COPD in BC.	service delivery	meso
Jatinderpreet Singh, Allison Lou, Michael Green, Erin Keely, Mary Greenaway and Clare Liddy	2021	Evaluation of an electronic consultation service for transgender care.	BMC Family Practice	champlain region, Ontario	mixed methods	This is a retrospective mixed methods analysis of the 62 eConsults. A descriptive analysis of time stamps for each eConsult was completed to assess the response time and total time spent by the specialist to complete the eConsult.An inductive and deductive content analysis of the primary care provider eConsult questions was also con-ducted to gain insight into common themes [12, 19, 22]. Two investigators, AL (transgender care specialist), and JPS (family medicine resident), independently reviewedclinical questions being asked by primary care provid-ers using a generalized validated taxonomy and also open coding to categorize questions not captured by the framework [23]. Both investigators discussed their independent assessments using an iterative approach to come to a consensus on a final list of themes. In many cases, a single eConsult had multiple questions, and thus, each eConsult was not restricted to only one theme to ensure no information was lost. In total, 20 eCon-sults were assessed until saturation was achieved. Once themes were identified, a single investigator (JPS) com-pleted a deductive analysis and went through and coded the remaining 42 eConsults. No new themes emerged from the final 42 eConsults. Themes were externally vali-dated by the remainder of the team (2 family physicians, 1 transgender specialist, and an endocrinologist).All eConsult close-out surveys (Fig. 1) done by pri-mary care providers were assessed to gain insight into the course of action taken following the consult, provider sat-isfaction, and to quantify the proportion of face to face specialist visits that were	Participants in the study included primary care providers who were registered to the Champlain BASE™ eConsult service and who initiated at least one electronic consul-tation between January 2017 and December 2018 to a transgender specialist	The aim of this study is to examine the impact of an electronic consultation service on improving access to transgender care as measured by the wait time and the number of traditional face-to-face referrals avoided. Also, this study aims to explore and characterize the content of clinical questions being asked to transgender specialists through eConsult.	eConsult	This study demonstrated that a transgender eConsult service has the potential to significantly improve access to care for transgender patients. Also, with one third of eConsults resulting in an avoided face to face refer-ral, this system may have the potential to free up space for more urgent cases that require a face-to-face refer-ral to be seen in a timely manner. Given the importance that timely access has on improving mental health and reducing suicide attempts, eConsult has the potential to make a substantial clinical impact on this population [2]. Furthermore, this study highlighted six key themes of eConsult questions that provide insight into poten-tial gaps in knowledge amongst primary care providers. This information could help in guiding future continuing education events or provide guidance in refining current guidelines to help address current knowledge gaps.Future studies will aim to study the value of a continu-ing education event guided by common gaps in transgen-der care identified through eConsult cases. Furthermore, it would be of interest to conduct patient and specialist interviews to understand their experiences with eConsult and to gain a better appreciation of the benefits of such a tool from their perspectives. Lastly, studying the impact of improved access to care through eConsult on clinical outcomes such as suicidal ideation and attempts will be examined in future work	service delivery	micro

Mary Helmer-Smith, Celeste Fung, Amir Afkham, Lois Crowe, Mohamed Gazarin, Erin Keely, Isabella Moroz, Clare Liddy	2020	The Feasibility of Using Electronic Consultation in Long-Term Care Homes.	Journal of American Medical Directors Association	champlain region, Ontario	mixed methods	Quantitative Data Collection: The eConsult service automatically collects use data from all cases,including patient age and gender, type of PCP (physician or nursepractitioner) submitting the case, PCP's practice location, specialtytype consulted (eg, dermatology), response interval, the specialists'self-reported billing time, and the PCP's responses to the mandatoryclose-out survey (Supplementary Material 1). Descriptive data aboutthe participating LTC homes (eg, number of long- and short-stay beds)were collected from the Ministry of Health and Long-Term Care'sofficial website.10Qualitative Data CollectionTen PCPs, 4 administrators, and 1 nurse champion from 2 LTChomes participated in a total of four 1-hour focus groups held be-tween September 5 and November 6, 2018. The objective of thesefocus groups was to explore the perspectives of early adopting PCPs onthe use of eConsult in LTC. The sessions were semi-structured andmoderated by the eConsult Research Team (Supplementary Material2). Transcripts of audio recordings from the focus groups were tran-scribed and uploaded into NVivo 11 (QSR International Pty Ltd) tofacilitate analysis.Quantitative Data AnalysisDescriptive statistics were calculated to characterize the case datafor analysis and evaluation. The number of long-stay beds in the re-gion was used as a proxy for population size when calculating thepopulation rate of eConsult in LTC.Qualitative Data AnalysisThree of the study authors (C.F., M.G., M.H.) analyzed the focusgroup transcripts using a constant comparative approach. Reviewersmet twice [illegible]	Ten PCPs, 4 administrators, and 1 nurse champion from 2 LTChomes + participants of Champlain BASE in Champlain Local Health Integration Network, a health region in Eastern Ontario, Canada	evaluate the feasibility of implementing eConsult in LTC	eConsult	Our study demonstrates that it is feasible to implement eConsult inLTC settings. Specialist response times and referral outcomes reflectedthose seen in primary care settings, and participants in the study'sfocus groups spoke positively of the service's value. Participantsidentified contextual factors affecting implementation in LTC settings,leading us to consider potential facilitators, such asfinancial in-centives, requirements for accreditation, and modernization of thedigital health infrastructure, including EMRs. More LTC homes shouldconsider adopting eConsult in order to improve access to specialistadvice for their residents. As the expanding population residing in LTCadvances in medical complexity and frailty, implementation ofeConsult provides an avenue to improve access to specialist care forour most vulnerable seniors.	service delivery	meso
Sarina R. Isenberg, Michael Bonares, Allison M. Kurahashi, Kavita Algu, and Ramona Mahtani	2022	Race and birth country are associated with discharge location from hospital: A retrospective cohort study of demographic differences for patients receiving inpatient palliative care	eClinical Medicine	Toronto	quantitative	The study used a retrospective cohort design based ondata obtained from a chart review of demographic datacollected as part of a regional health equity initiativeand clinical data available in the electronic medicalrecord at Mount Sinai Hospital in Toronto.	We included all patients referred to and seen by theinpatient palliative care team at Mount Sinai Hospitalin Toronto, Canada between April 1, 2018 to March 31,2019. We excluded patients who were referred to pallia-tive care but did not receive a consultation as thesepatients typically have inappropriate referrals, die beforebeing seen, or wish to not receive palliative care. We alsoexcluded those who did not have a completed demo-graphic form available in their chart. Our sample sizewas based on how many patients had completed thedemographic form during the timeframe of interest,which was a hospital-wide initiative distinct from theresearch study. We did not [illegible]	First, we describedcommonly used demographic variables among a cohortof patients in a tertiary hospital receiving specializedinpatient palliative care consultation. Second, we evalu-ated differences in clinical process indicators (i.e., dis-charge location, length of stay (LOS), and timing ofinpatient palliative care referral and consultation) whenstratified by these demographic characteristics	EMR	Our study found that, in a cohort of patients receiv-ing inpatient palliative care consultations, variables sig-nificantly associated with disposition were birth countryand race/ethnicity. Language was significantly associ-ated with time from admission to palliative care referral.That said, there are several limitations with our studythat suggest further research is needed. These prelimi-nary findings suggest inequalities may exist in palliativecare and point to specific areas that warrant a morerobust analysis. The few possible inequalities we foundneed further study using larger sample sizes and multi-variate models. Addressing inequalities will need fur-ther studies that focus on understanding the underlyingpractices that constructed and maintained theseinequalities in care. There is additional value to thisstudy as few health administrative databases containdetailed demographic data. Since the hospital had thespecial initiative to collect demographic data frompatients, we were able to explore the relationshipbetween these characteristics and various outcomes.Hopefully this paper helps to further the case for theprospective collection of this data and its ability to helpus to identify health disparities.	service delivery	meso
Fiorella A. Heald, Susan Marzolini, Tracey J. F. Colella, Paul Oh, Rajni Nijhawan and Sherry L. Grace	2021	Women's outcomes following mixed-sex, women-only, and home-based cardiac rehabilitation participation and comparison by sex	BMC Women's Health	Toronto	quantitative	This was a retrospective cohort study, with 4 compari-son groups. Data used in this study were extracted from an electronic patient management record utilized across the University Health Network (UHN) Cardiovascular Prevention and Rehabilitation sites located in Toronto, Ontario, Canada, from January 1, 2017 to February 28, 2020 (only to March 31, 2019 for Toronto Western site as the program was shortened to 4 months at that time due to the wait list); data were extracted from pre and post-program.	Study-specific inclusion criteria were graduated female patients who attended at least 1 on-site exercise session for supervised models and 1 telephone consultation for the home-based model; males who were in the super-vised model were eligible for matching. Patients were considered to have completed the program if they did not fail to attend 2–3 consecutive CR sessions (telephone consultations for home-based model) without notice and respond to communication attempts made by the pro-gram, and completed the post-program assessments	The objectives of this study were to compare: (1) cardiorespiratory fitness, (2) risk factors (i.e., blood pressure, lipids, tobacco use, and anthropo-metrics), and (3) psychosocial well-being (i.e., depres-sive symptoms and quality of life), in women between the three CR models.	EMR	Participants in mixed-sex only achieved significant improvements in HDL-C, waist circumference, quality of life, and depressive symptoms by program end; Fitness was significantly greater with mixed-sex than women-only. The study was under-powered to make comparisons to home-based, but improvements in cardiorespiratory fitness require more study. Whether fully gender-tailored programs are advantageous requires more controlled, large-scale investigation. We need to better engage women in all models of CR, to ensure they achieve opti-mal outcomes	service delivery	micro

Nicholas James Schubert, Paul J. Backman, Rajiv Bhatla, Kimberly M. Corace	2019	Telepsychiatry and patient-provider concordance.	CJRM	ontario	quantitativ e	Patient and provider questionnairesThe patient questionnaire contained a short demographic section. Both the provider and the patient questionnaires were designed to measure the perceptions of access and satisfaction with telepsychiatry sessions. The items in the questionnaires were adapted from other published surveys used in similar evaluations of telepsychiatry and were selected to capture important aspects of the patient experience with telepsychiatry consultations. Each item used a 5-point Likert response scale, with responses ranging from 'strongly disagree' to 'strongly agree'.16-18Data analysisAnalyses were performed using SPSS software version 24 (IBM Corp., Armonk, NY, USA).19 Patient and provider responses to survey items were summarised with frequency counts and percentages. Bennett et al.'s coefficient S was used as the index of concordance between patients' and providers' responses to paired survey items.20S yields values ranging from -1, representing absolute discordance, to 1, representing absolute concordance, with value of 0 representing the proportion of concordance that would be expected by chance based on the number of response categories. The 5-point response scale used in the survey was collapsed to three response categories (i.e. "disagree', 'neither agree nor disagree' and 'agree') prior to calculating S. The statistical level of significance was set at P < 0.05.	Participants were outpatients who attended telepsychiatry consultation appointments between February and October 2016 from one of the rural partner sites and providers were those who provided consultations from the tertiary academic site. The current study was conducted at a tertiary academic site in Ottawa with an on-site telepsychiatry programme along with rural primary healthcare sites located throughout Eastern Ontario. The partner sites were situated at a minimum distance of 65 km and a maximum distance of 200 km from the tertiary academic site. Partner sites were selected to be evenly distributed throughout the region, and they were screened to ensure [illegible]	the objectives of this study were twofold which are described as follows: the primary objective was to assess patient and provider perceptions of telepsychiatry (i.e., access to care and satisfaction with the service) in a Canadian sample, and the secondary objective was to assess the level of concordance between patients and providers.	telehealth	This research demonstrates high levels of satisfaction and concordance between patients and providers concerning telepsychiatric consultations provided from an urban mental health centre to rural regional clinics. These high levels of satisfaction among both patients and providers indicate that our regional telepsychiatry consultation model is one which other urban mental health centres located within similar catchment areas could consider for potential application to their context. The high level of concordance also suggests that good adherence and patient outcomes may be achievable within the telepsychiatry consultation model. These results provide support for the use of telepsychiatry consultations to improve access to psychiatric care for rural populations.	service delivery	micro
Saagar Walia, Dalton Wolfe, David Keast, Chester Ho, Karen Ethans, Scott Worley, Colleen O'Connell, and Denise Hill	2019	Facilitators and Barriers for Implementing an Internet Clinic for the Treatment of Pressure Injuries.	Telemedicine and eHealth	Canada	mixed methods	This study was conducted at four Canadian centers: (1)Foothills Medical Centre (Calgary, AB), (2) Parkwood Institute(London, ON), (3) Stan Cassidy Centre for Rehabilitation(Fredericton, NB), and (4) Winnipeg Health Sciences Centre(Winnipeg, MB). Each site had a lead, coordinator, and in-terdisciplinary team that worked collaboratively with theprinciple investigator and project coordinator. The interdis-ciplinary team composition varied across sites and could in-clude physicians (family physician or specialized inphysiatry), nurses, occupational therapists, physiotherapists,registered dietitians, and home care nurses.Each site's interdisciplinary team identified components oftheir standard clinical care pathway and determined how theycould be integrated with the study technologies. Each inter-disciplinary team developed a set of clinical assessments thatwould be completed by the patient or their caregiver. Beforebeginning recruitment, all team members completed trainingwith study technologies.Upon participant enrolment, a standardized clinical andstudy protocol was administered.1. Initiation: The site coordinator visited the participant'shome to set up a webcam and install antivirus protectionon the participant's personal computer. The participantcompleted a tutorial on how to use the patient website andwebcam for videoconferencing. After this visit, partici-pants were asked to complete their initial assessment.2. In-Home Assessment: After the participants completedtheir initial assessment, the home care nurse visited [illegible]	This study was conducted at four Canadian centers: (1)Foothills Medical Centre (Calgary, AB), (2) Parkwood Institute(London, ON), (3) Stan Cassidy Centre for Rehabilitation(Fredericton, NB), and (4) Winnipeg Health Sciences Centre(Winnipeg, MB). Participants and clinicians com-pleted satisfaction and self-confidence surveys	This study, conducted at four institutions across Canada,aimed to enhance access to a specialized interdisciplinary teamthrough the creation of an internet clinic addressing the treat-ment of PIs in persons with SCI. The intent was to deliver theseservices directly into the participant's home, thereby reducingthe need for in-person clinic visits. The primary objective of thestudy was to assess the feasibility of this model of care.	telehealth	This study highlighted the need for continuing educationfor home care nurses. In some cases, home care nurses did nothave specialized knowledge or expertise in caring for personswith SCI with a PI. This is understandable given that homecare nurses provide care for a number of patients with dif-ferent health conditions. Feedback from specialist care pro-viders in this study suggested that providing education tocommunity providers could enhance care in the community.A telehealth approach necessitates that home care nurses tohave specialized knowledge to properly assess (e.g., examinerisk factors, measure the PI, and capture photographs) andmanage (e.g., clean and dress) the PI. This is necessary giventhat assessment information is required for the specialist teamto develop a treatment plan and the patient will not be seen in-person. In this study, some education was provided live duringvideoconferences; however, some aspects of care would bebest learned through in-person training.There is also evidence that a telehealth approach can beutilized beyond PI management to address other secondarycomplications (bladder management, pain, etc.) as well.26Amodel of care and telehealth system that adopts a broad ap-proach to health may lead to economies of scale and synergiesin training and capacity building. Future studies could ex-amine optimal telehealth models of care that could be utilizedto address a number of secondary complications for personswith SCI.25	service delivery	meso
Priscilla Ferrazzi and Terry Krupa	2018	Remoteness and its impact on the potential for mental health initiatives in criminal courts in Nunavut, Canada.	International Journal of Polar Health	Nunavut	qualitative	A total of 55 semi-structured interviews were con-ducted by the principal investigator (first author) in 2013with the assistance of Inuit research assistants andInuktitut-speaking interpreters as needed. Recruitmentof participants reflected purposeful sampling. Interviewswere generally held in community organisation facilities,government offices, at the Nunavut Research Institute,and on occasion in peoples'homes. Forty-eight interviewswere audio-recorded and transcribed verbatim by certi-fied court reporters and 7 interviews were recorded usingfield notes. The principal investigator returned toparticipants to confirm that a summary of their raw inter-view data reflected their intended meaning in circum-stances where interviews involved interpreters, whereparticipants struggled with the English language andwhere interviews were recorded using field notes.Interviews involved the justice sector (e.g. judges,defence lawyers, prosecutors and police), the healthsector (e.g. psychiatrists and nurses), community organi-sations (e.g. hamlet officials and members of Inuit organi-sations) and community members (e.g. elders and carersof people affected by mental illness). Among the totalinterviews, 13 were with"fly-in"participants (e.g. judges,defence lawyers, prosecutors and psychiatrists) who ser-vice Iqaluit and the rest of the territory by plane and playcritical health and justice roles not otherwise available inoutlying communities. As these fly-in interviews wererelevant to all 3 communities, they comprised a compo-nent of the data for each. Accordingly, a total of 26 inter-[illegible]	The study involved 3 communities with different healthand justice resource capacities and relative remoteness.Iqaluit, Arviat and Qikiqtarjuaq represented high, med-ium and low capacities. Recruitmentof participants reflected purposeful sampling	This research uses the 3 principles of TJ, and theirassociated 18 objectives, to explore how geographicremoteness is perceived to affect the potential for crim-inal court mental health initiatives in Nunavut. Thestudy examines perceptions by key health, justice andcommunity stakeholders concerning the likely impactsof remoteness as well as the potential role of remotecommunication technologies.	telehealth	Criminal court mental health initiatives and the thera-peutic aims of TJ offer Nunavut courts the potential fora rehabilitation-oriented response to people affected bymental illness. Results from this research, however, sug-gest that remoteness–a profound realty in Nunavut'sArctic communities–is considered by many justice,health and community stakeholders as both an existingchallenge to criminal court responses to peopleaffected by mental illness and a significant considera-tion in potential efforts to introduce criminal courtmental health initiatives in the territory. Indeed, percep-tions of remoteness-related impacts, including delay,and scepticism by the justice sector about remote tech-nologies are affecting decisions by lawyers that, evennow, reduce the likelihood that accused peopleaffected by mental illness will pursue mental healthavenues prescribed by law and therapeutic optionsover regular court processing. That is, more peoplewith mental illness may be facing criminal sanctionswithout assessment and treatment. Efforts to changethe current circumstance are needed before criminalcourt mental health initiatives in the territory are likelyto succeed. More research is needed to understand theroot of these perceptions and approaches to amelioratethem. This work would go a long way to understandingwhat needs to be done to mitigate geographic remote-ness as a factor affecting the potential for criminal courtmental health initiatives in Nunavut.	service delivery	meso

Rebecca A. Chedid, Rowan M. Terrell, Karen P. Phillips	2018	Best practices for online Canadian prenatal health promotion: A public health approach.	Women and Birth	Canada	quantitative	Evaluation of pre natal health promotion content on government-hosted websites and pre natale-classes began in September, 2016 and was completed in May, 2017, with all websites periodically revisited throughout this time frame. Evaluation grids were created to assess pre natal health content based on four main criteria: comprehensiveness, evidence-based information, accessibility and inclusivity. Each website and pre natale-class was evaluated in a standardised manner by at least two researchers, using a scoring system described below. Scoring discrepancies were discussed in team meetings with final scores agreed upon by consensus. Scores are reported as the average score per jurisdiction, with atypical findings described in the results. Parenting/pregnancy portals or the home page of the host website were evaluated followed by keyword searches in English and French; corresponding to terms identified in Box1. Content was determined to be pre natal health promotion if the information was targeted to lay audiences; used non-expert language and promoted pre natal health information and resources. Scientific articles; technical bulletins or information specifically targeted to health professionals/researchers were not considered health promotion content; and thus were not evaluated. Pre natale-classes were reviewed in their entirety including the completion of online modules and tests; as well as reviewing videos and other content—similar to the experiences of regular class participants.	Six commercially-developed prenatale classes endorsed and promoted bypublic health regional units and one prenatale-class developed by an Ontario public health unit (Durham Region Health Department) were included in this study	This study will assess whether government-hosted websites and affiliated online prenatal education programmes (prenatale-classes) provide accessible, inclusive, comprehensive and evidence-based prenatal health promotion to Canadians	telehealth	In conclusion, Canadian government-hosted websites and pre natale-classes generally provided comprehensive and evidence-based pre natal health promotion. Major gaps in online pre natal health promotion included the availability of bilingual and multilingual resources, representations of non-traditional families, including LGBTQ parents, and lack of specialised pregnancy information targeted to women living with disabilities and communities of Indigenous peoples and immigrants. Organisations are encouraged to collaborate with other jurisdictions and community stakeholders to facilitate the exchange of online resources and materials, ensuring visual representation of our heterogeneous community of pregnant women, and the availability of evidence-based, comprehensive, accessible and inclusive pre natal health promotion.	service delivery	meso
Alex Zhou, Abdelhady Osman, Genesis Flores, Dhuvaraha Srikrishnaraj, Jayashree Mohanty, Retage Al Bader, Amy Llancari, Aya El-Hashemi, Manahel Elias, Kanza Mirza, Maureen Muldoon,Ryan Palazzolo, Farwa Zaib, Indryas Woldie, and Caroline Hamm	2023	Critical Illness in Migrant Workers in the Windsor-Essex Region: A Descriptive Analysis	International Journal of Environmental Research and Public Health	Windsor-essex region	qualitative	data were collected through a combination of a retrospective chart review andpatient interviews.	migrant workers at Windsor Regional Hospital(WRH)	Our objective was to collect informationon the experiences of migrant workers experiencing a critical illness at Windsor Regional Hospital(WRH) between 31 December 2011 and 31 December 2021	EMR	Our chart review and interviewsfound that critically ill migrant workers generally receive medical care congruent with theCanadian standard of care while in Canada. Our study highlights medical repatriation as a persistent concern for critically ill migrantworkers in the Windsor-Essex region. This supports the existing knowledge of repatriationas an often unique determinant of health for migrant workers across Canada. Specifically, the findingsof this study highlight broader issues around the continuation of healthcare coverage forvulnerable populations. Until public health policy changes are enacted, medical repatriationand a lack of coverage remain obstacles to the Canadian healthcare principles of universalityand accessibility while impinging on patient autonomy.	service delivery	micro
June C. Carroll, Clare	2022	Use of eConsult to enhance genetics service delivery in primary	Genetics in	Ontario,	mixed	PCP questionnaires regarding eConsult's utility,	2 regions of Ontario,	Our purpose was to	eConsult	eConsult offers a potential solution for receiving timely	service delivery	meso
Clare Liddy and Erin Keely	2018	Using the Quadruple Aim Framework to Measure Impact of Heath Technology Implementation: A Case Study of eConsult.	Journal of the American Board of Family Medicine	eastern Ontario	qualitative	A descriptive overview of data was collected between April 1, 2011, and August 31, 2017,using 4 dimensions of care outlined by the Quadruple Aim Framework: patient experience, providerexperience, costs, and population health. Findings were drawn from use data, primary care providercloseout surveys, surveys/interviews with patients and provider, and costing data	Champlain Local Health Integration Network (LHIN), the health region where eConsult is administered and where it be-gan as a regional service. The Champlain LHIN is situated in eastern Ontario, Canada	In this article, we describehow to evaluate the impact of a health technology innovation through the 4 dimensions of care outlinedby the Quadruple Aim Framework, using our experience with the Champlain Building Access to Special-ists through eConsultation (BASE) eConsult service as a case example	eConsult	The eConsult service has been widely adopted in our region and is currently expandingto new jurisdictions across Canada. However, although we successfully demonstrated eConsult's impacton patient experience, provider satisfaction, and reducing costs, we met several challenges in evaluatingits impact on population health. More work is needed to evaluate eConsult's impact on key populationhealth metrics (eg, mortality, morbidity, and system use). Efforts to conduct such evaluations are under-way.	service delivery	meso
Justin Joschkoa, Clare Liddy, Isabella Moroza, Marnie Reichec, Lois Crowea, Amir Afkham, and Erin Keely	2018	Just a click away: exploring patients' perspectives on receiving care through the Champlain BASETM eConsult service.	Family practice	eastern Ontario	qualitative	We conducted a thematic analysis of patient interviews using a constant comparative approach. Patients whose primary care providers used the eConsult service in their care were contacted by telephone between June 2015 and January 2016 and completed 15-min semi-structured interviews.	The Champlain BASE (Building Access to Specialists through eConsultation) eConsult service is located in the Champlain health region of Eastern Ontario, Canada	We interviewed patients who were treated using eConsult in order to explore their attitudes towards the service and their experiences of receiving care via the service	eConsult	Patients expressed acceptance for eConsult as a model for improving access to specialist care, had largely positive experiences with it as a model of care delivery, and supported its use in their future care.	service delivery	micro
