# Supplementary material for: Artificial intelligence and social accountability in the Canadian health care landscape: A rapid literature review
Source: PLOS Digit Health. 2024 Sep 12;3(9):e0000597. doi: 10.1371/journal.pdig.0000597 (PMC11392241; doi:10.1371/journal.pdig.0000597)
Supplement: S3 File — (PDF) [file pdig.0000597.s003.pdf]

Supplemental File 3

Keywords and Search Strategy

KEYWORDS

Search Concepts:

1. Social Accountability

Keywords searched: (**Social responsibility, Health Equity, Bias, DIVERSITY, EQUITY, INCLUSION, Cultural Diversity, Health Inequities, Healthcare Disparities, Health Services Accessibility, Health Status Disparities, Socioeconomic Factors, Social Determinants of Health, Gender Equity**)

2. Artificial Intelligence

Keywords searched: (**Artificial intelligence**, **Machine learning, Deep learning, Natural language processing, Neural Network, Computer**, **Expert Systems**, **DECISION MAKING, COMPUTER-ASSISTED**, **Decision Support Systems, Clinical, Robotics**, **Robotic Surgical Procedures, Surgery, Computer-Assisted, Data Mining, Diagnosis, Computer-Assisted**, **Therapy, Computer-Assisted, Drug Therapy, Computer-Assisted, Forecasting**, **Telemedicine**, **Wearable Electronic Devices**, **Precision Medicine**, **Theranostic Nanomedicine, Nanomedicine, Medical Informatics, Electronic Health Records**.)

3. Location

Keywords searched: (**Canada**)

SEARCH STRATEGY

MEDLINE

Ovid MEDLINE(R) and Epub Ahead of Print, In-Process, In-Data-Review & Other Non-Indexed Citations, Daily and Versions <1946 to September 15, 2023>

1 exp *Artificial Intelligence/ 109627

2 (artificial* adj2 intelligen*).mp. [mp=title, book title, abstract, original title, name of substance word, subject heading word, floating sub-heading word, keyword heading word, organism supplementary concept word, protocol supplementary concept word, rare disease supplementary concept word, unique identifier, synonyms, population supplementary concept word, anatomy supplementary concept word] 62204

3 exp Medical Informatics/ or exp Decision Making, Computer-Assisted/ or exp Diagnosis, Computer-Assisted/ or exp Therapy, Computer-Assisted/ or exp Decision Support Techniques/ or Data Mining/ or Decision Support Systems, clinical/ 500124

4 exp Surgery, Computer-Assisted/ 38637

5 Forecasting/ 91758

6 exp Telemedicine/ 45299

7 exp Nanomedicine/ 11504

8 Precision Medicine/ 26684

9 exp Wearable Electronic Devices/ 19132

10 exp Medical Records Systems, Computerized/ 48723

11 1 or 2 or 3 or 4 or 5 or 6 or 7 or 8 or 9 or 10 838966

12 exp Canada/ 182795

13 Social Responsibility/ 20582

14 (social* adj3 account*).mp. [mp=title, book title, abstract, original title, name of substance word, subject heading word, floating sub-heading word, keyword heading word, organism supplementary concept word, protocol supplementary concept word, rare disease supplementary concept word, unique identifier, synonyms, population supplementary concept word, anatomy supplementary concept word] 3616

15 exp Racism/ 6628

16 exp Race Factors/ 1193

17 Community Participation/ 18504

18 (communit* adj3 engag*).mp. [mp=title, book title, abstract, original title, name of substance word, subject heading word, floating sub-heading word, keyword heading word, organism supplementary concept word, protocol supplementary concept word, rare disease supplementary concept word, unique identifier, synonyms, population supplementary concept word, anatomy supplementary concept word] 10865

19 Intersectoral Collaboration/ 2580

20 Stakeholder Participation/ 2113

21 exp Human Rights/ 156190

22 Civil Rights/ 10183

23 exp Social Problems/ 352763

24 exp Vulnerable Populations/ 12845

25 Gender Equity/ 608

26 "Social Determinants of Health"/ 6606

27 exp Socioeconomic Factors/ 513375

28 exp Healthcare Disparities/ 22307

29 Health Services Accessibility/ 85779

30 13 or 14 or 15 or 16 or 17 or 18 or 19 or 20 or 21 or 22 or 23 or 24 or 25 or 26 or 27 or 28 or 29 1041953

31 11 and 12 and 30 1367

32 limit 31 to yr="2018 - 2023" 253

NOTE 1: Limiting by date range and by language (English): 251 results (not meaningful enough to bother)

NOTE 2: Did not include the following terms (exp Education, Medical; Biomedical Research, Health Services Research) because it drastically reduced the final article count.

Link to search results in RefWorks: https://refworks.proquest.com/public-share/xxtuL1ojsz8iPzzlaCqGX8XTIfRzLRbZVzxv7TSGzhC4

RR_AI_EMBASE 09 18 2023

Embase <1980 to 2023 Week 37>

1 exp *artificial intelligence/ 40951

2 (artificial* adj2 intelligen*).mp. [mp=title, abstract, heading word, drug trade name, original title, device manufacturer, drug manufacturer, device trade name, keyword heading word, floating subheading word, candidate term word] 71484

3 medical informatics/ 23250

4 computer assisted diagnosis/ 41509

5 computer assisted therapy/ or computer assisted drug therapy/ 5749

6 "prediction and forecasting"/ 18763

7 telemedicine/ 44795

8 exp nanomedicine/ 19622

9 personalized medicine/ 71943

10 exp wearable computer/ 8886

11 electronic medical record/ 79931

12 data mining/ 18391

13 computer assisted surgery/ or robot assisted surgery/ 34022

14 decision support system/ or clinical decision support system/ 32960

15 1 or 2 or 3 or 4 or 5 or 6 or 7 or 8 or 9 or 10 or 11 or 12 or 13 or 14 446993

16 exp Canada/ 209711

17 social responsibility/ 3400

18 (social* adj3 account*).mp. [mp=title, abstract, heading word, drug trade name, original title, device manufacturer, drug manufacturer, device trade name, keyword heading word, floating subheading word, candidate term word] 4031

19 exp racism/ 14454

20 race/ 82703

21 community participation/ 4314

22 (communit* adj3 engag*).mp. [mp=title, abstract, heading word, drug trade name, original title, device manufacturer, drug manufacturer, device trade name, keyword heading word, floating subheading word, candidate term word] 12879

23 intersectoral collaboration/ 4141

24 stakeholder engagement/ 8198

25 exp human rights/ 306627

26 civil rights/ 7375

27 exp social problem/ 1393231

28 exp vulnerable population/ 27732

29 gender equity/ 1556

30 "social determinants of health"/ 19789

31 exp socioeconomics/ 1315564

32 health disparity/ 34920

33 health care access/ 87177

34 17 or 18 or 19 or 20 or 21 or 22 or 23 or 24 or 25 or 26 or 27 or 28 or 29 or 30 or 31 or 32 or 33 2883698

35 15 and 16 and 34 809

36 limit 35 to yr="2018 - 2023" 426

NOTE 1: Limiting by date range and by language (English): 425 results (not meaningful enough to bother)

NOTE 2: Did not include the following terms (exp Medical Education; Medical Research, Health Services Research) because it drastically reduced the final article count.

Link to results in RefWorks: https://refworks.proquest.com/public-share/jQiqt6hiDhxNetXa2JhkLrfTEZkw6EejfIvnErPt2ogv
