# Supplementary material for: Artificial intelligence and social accountability in the Canadian health care landscape: A rapid literature review
Source: PLOS Digit Health. 2024 Sep 12;3(9):e0000597. doi: 10.1371/journal.pdig.0000597 (PMC11392241; doi:10.1371/journal.pdig.0000597)
Supplement: S4 File — (PDF) [file pdig.0000597.s004.pdf]

Name	Relevancy: Meets inclusion and exclusion criteria, Acceptable format, Current	Reliability: Assess for any missing data: methods, results, conclusions; Assess for conflicts of interest; Assess for funding source; Determine if reviewed by ethics board, how informed consent was obtained (for primary studies)	Validity: Internal validity; External validity; Bias; ensure results support authors conclusions	Applicability: Lessons from the sources reviewed can be applied to our context	CASP checklist	Notes
The Preferences of Transgender and Nonbinary People for Virtual Health Care After the COVID-19 Pandemic in Canada: Cross-sectional Study	☑	☑	☑	☑	☑	
Using the quadruple aim to understand the impact of virtual delivery of care within Ontario community health centres: a qualitative study	☑	☑	☑	☑	☑	Can't tell: Has the relationship between researcher and participants been adequately considered?
Sociodemographic Differences in Physician-Based Mental Health and Virtual Care Utilization and Uptake of Virtual Care Among Children and Adolescents During the COVID-19 Pandemic in Ontario, Canada:A Population-Based Study	☑	☑	☑	☑	n/a	
Health equity related challenges and experiences during the rapid implementation of virtual care during COVID-19: a multiple case study	☑	☑	☑	☑	☑	
Describing a complex primary health care population in a learning health system to support future decision support and artificial intelligence initiatives	☑	☑	☑	☑	☑	
Co-Design to Support the Development of Inclusive eHealth Toolsfor Caregivers of Functionally Dependent Older Persons: Social Justice Design	☑	☑	☑	☑	☑	
Rural use of health service and telemedicine during COVID-19: The role of access and eHealth literacy	☑	☑	☑	☑	☑	
The perspective of Canadian health care professionals on abortion service during the COVID-19 pandemic	☑	☑	☑	☑	☑	Can't tell: Has the relationship between researcher and participants been adequately considered?
Assessing Virtual Mental Health Access for Refugees duringthe COVID-19 Pandemic Using the Levesque Client-CenteredFramework: What Have We Learned and How Will We Plan forthe Future?	☑	☑	☑	☑	☑	Can't tell: Has the relationship between researcher and participants been adequately considered?
Sociodemographics Associated With Risk of Diabetic Retinopathy Detected by Tele-Ophthalmology: 5-Year Results of the TorontoTele-Retinal Screening Program	☑	☑	☑	☑	☑	
Improving value and access to specialty medical care for families: a pediatric surgery telehealth program	☑	☑	☑	☑	☑	Can't tell: Has the relationship between researcher and participants been adequately considered?

Essential requirements for establishing and operating data trusts: practical guidance co-developed by representatives from fifteen canadian organizations and initiatives	☑	☑	☑	☑	☑	Can't tell as recruitment was not talked about: Was the recruitment strategy appropriate to the aims of the research? & Can't tell: Has the relationship between researcher and participants been adequately considered?
Variability in patient sociodemographics, clinical characteristics, and healthcare service utilization among 107,302 treatment seeking smokers in Ontario: A cross-sectional comparison	☑	☑	☑	☑	n/a	
Perspectives on delivering safe and equitable trauma-focused intimate partner violence interventions via virtual means: A qualitative study during COVID-19 pandemic.	☑	☑	☑	☑	☑	Can't tell: Has the relationship between researcher and participants been adequately considered?
Correlates of past year suicidal thoughts among sexual and gender minority young adults: A machine learning analysis.	☑	☑	☑	☑	n/a	
Patient perceptions of the benefits and barriers of virtual postnatal care: a qualitative study.	☑	☑	☑	☑	☑	
Key factors for national spread and scale-up of an eConsult innovation	☑	☑	☑	☑	☑	Can't tell: Has the relationship between researcher and participants been adequately considered?
Best practices for EHR implementation: A BC First Nations community's experience	☑	☑	☑	☑	☑	
Characterizing the Use of Telepsychiatry for Patients with Opioid Use Disorder and Cooccurring Mental Health Disorders in Ontario, Canada	☑	☑	☑	☑	☑	
Health, Social, Education, and Justice Outcomes of Manitoba First Nations Children Diagnosed with Fetal Alcohol Spectrum Disorder: A Population-Based Cohort Study of Linked Administrative Data	☑	☑	☑	☑	☑	
Deliver Cardiac Virtual Care: A Primer for Cardiovascular Professionals in Canada	☑	☑	☑	☑	n/a	
Improving Access to Automated External Defibrillators in Rural and Remote Settings: A Drone Delivery Feasibility Study	☑	☑	☑	☑	n/a	
Beliefs, attitudes and experiences of virtual overdose monitoring services from the perspectives of people who use substances in Canada: a qualitative study	☑	☑	☑	☑	☑	Can't tell: Has the relationship between researcher and participants been adequately considered?
Disparities in self-reported healthcare access for airways disease in British Columbia, Canada, during the COVID-19 pandemic. Insights from a survey co-developed with people living with asthma and chronic obstructive pulmonary disease.	☑	☑	☑	☑	n/a	
Evaluation of an electronic consultation service for transgender care.	☑	☑	☑	☑	n/a	

The Feasibility of Using Electronic Consultation in Long-Term Care Homes.	☑	☑	☑	☑	n/a	
Race and birth country are associated with discharge location from hospital: A retrospective cohort study of demographic differences for patients receiving inpatient palliative care	☑	☑	☑	☑	☑	
Women's outcomes following mixed-sex, women-only, and home-based cardiac rehabilitation participation and comparison by sex	☑	☑	☑	☑	☑	
Telepsychiatry and patient-provider concordance.	☑	☑	☑	☑	n/a	
Facilitators and Barriers for Implementing an Internet Clinic for the Treatment of Pressure Injuries.	☑	☑	☑	☑	n/a	
Remoteness and its impact on the potential for mental health initiatives in criminal courts in Nunavut, Canada.	☑	☑	☑	☑	☑	Can't tell: Has the relationship between researcher and participants been adequately considered?
Best practices for online Canadian prenatal health promotion: A public health approach.	☑	☑	☑	☑	n/a	
Critical Illness in Migrant Workers in the Windsor-Essex Region: A Descriptive Analysis	☑	☑	☑	☑	☑	Can't tell: Has the relationship between researcher and participants been adequately considered?
Use of eConsult to enhance genetics service delivery in primary care: A multimethod study.	☑	☑	☑	☑	n/a	
Using the Quadruple Aim Framework to Measure Impact of Heath Technology Implementation: A Case Study of eConsult.	☑	☑	☑	☑	☑	Can't tell: Has the relationship between researcher and participants been adequately considered?
Just a click away: exploring patients' perspectives on receiving care through the Champlain BASETM eConsult service.	☑	☑	☑	☑	☑	Can't tell: Has the relationship between researcher and participants been adequately considered?
